# Screening and Identification of Novel DNA Aptamer for Targeted Delivery to Injured Podocytes in Glomerular Diseases

**DOI:** 10.1002/advs.202412356

**Published:** 2025-04-03

**Authors:** Chao Zhou, Zhaofeng Luo, Zheng Zhang, Qing Ye, Dongjie Wang, Hanyan Meng, Jiayu Zhang, Shifan Zhu, Lidan Hu, Jianhua Mao

**Affiliations:** ^1^ Department of Nephrology Children's Hospital National Clinical Research Center for Child Health Zhejiang University School of Medicine Hangzhou 310052 China; ^2^ Liangzhu Laboratory Zhejiang University School of Medicine Hangzhou 310058 China; ^3^ The Key Laboratory of Zhejiang Province for Aptamers and Theranostic Aptamer Selection Center Hangzhou Institute of Medicine (HIM) Chinese Academy of Sciences Hangzhou Zhejiang 310022 China

**Keywords:** aptamer, nephropathy, podocyte, targeting delivery

## Abstract

Selective drug delivery to podocytes remains a challenge. Aptamers, nucleic acids that bind specific cells, offer a potential solution, though podocyte‐targeting aptamers have not yet been developed. Podocytes stimulated with adriamycin, puromycin aminonucleoside, and high glucose are used to screen an single‐stranded DNA (ssDNA) library (10¹⁵ sequences). High‐throughput sequencing identifies nucleotide sequences, and the aptamer's affinity, stability, cytotoxicity, uptake, biodistribution (especially to podocyte), target protein and ability to deliver siRNA are evaluated. After 11–14 rounds of selection, high‐affinity pools are identified. Sequencing reveals 23,848 unique sequences, narrowed down to 12 candidates. Aptamer S7 is specifically bound to podocytes, and its truncated version, RLS‐2, demonstrates superior affinity (50–70 nM) and improved stability with phosphorothioate modifications. RLS‐2 exhibits no significant cytotoxicity, is internalized by podocytes, and localized to lysosomes. In adriamycin‐induced and diabetic nephropathy mice, RLS‐2 preferentially accumulates within glomeruli. Its specificity to podocyte is verified by colocalization examination and quantitated via flowcytometry. EPB41L5 is identified as a target protein. Aptamer‐siRNA chimeras based on RLS‐2 successfully downregulate gene expression without the need for transfection reagents in vitro. These findings underscore the potential of RLS‐2 as a promising agent for the development of podocyte‐targeted drug delivery systems.

## Graphical abstract

This study develops RLS‐2, a high‐affinity aptamer that specifically targets injured podocytes, making it a promising candidate for targeted drug delivery. In vitro, RLS‐2 shows stability, specificity, efficient siRNA delivery via AsiCs, and uptake by podocytes. In vivo, it preferentially accumulates in the glomeruli of nephropathy models, supporting its potential for podocyte‐targeted therapies.

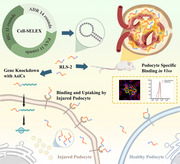

## 1 Introduction

Podocyte damage plays a critical role in the progression of proteinuria‐related diseases and is a leading contributor to end‐stage kidney disease (ESKD),^[^ ^1^ ^]^ a condition increasingly burdening healthcare systems worldwide.^[^ ^2^ ^]^ The excessive stress on podocytes can lead to structural damage, including effacement, and detachment, ultimately resulting in their loss,^[^ ^3^ ^]^ and the exacerbation of nephrotic syndrome, manifested as proteinuria, hypoalbuminemia, hyperlipidemia, and heightened infection risks.^[^ ^4^ ^]^ Given the limited regenerative capacity of these highly differentiated cells, it is crucial to mitigate podocyte injury to improve treatment outcomes. Targeting podocytes for drug delivery offers a promising therapeutic strategy. Drugs such as glucocorticoids, renin‐angiotensin system (RAS) inhibitors, and immunosuppressants have shown direct protective effects on podocytes, underscoring the importance of targeted drug delivery in enhancing therapeutic efficacy while minimizing side effects.^[^ ^5^, ^6^ ^]^ Additionally, gene therapy holds the potential for treating hereditary podocytopathies by addressing underlying genetic causes.^[^ ^7^ ^]^ However, the challenge of achieving effective and selective delivery to podocytes remains a critical obstacle in current therapeutic approaches.

Despite advancements in renal targeting research, most studies have focused on tubular, endothelial, and mesangial cells, with relatively few dedicated to podocytes.^[^ ^8^ ^]^ While some studies have successfully demonstrated podocyte‐targeting vectors in animal models,^[^ ^9^, ^10^ ^]^ limitations such as insufficient in vivo validation^[^ ^11^, ^12^ ^]^ and off‐target effects outside the kidneys^[^ ^13^ ^]^ persist. Moreover, antibodies used in some targeting strategies carry risks of glomerular injury, further emphasizing the need for safer and more efficient drug carriers for podocytes.

Aptamers, single‐stranded nucleic acid molecules that fold into 3D structures, offer a viable alternative to antibodies, with advantages such as low immunogenicity, ease of modification, and lower synthesis costs. Consequently, aptamers have been extensively investigated for targeting tumor cells in recent years.^[^ ^14^ ^]^ In addition, for kidney diseases, some aptamers have also demonstrated renal protective effects in various animal models of nephropathy. For instance, aptamers targeting PDGF‐B,^[^ ^15^, ^16^, ^17^ ^]^ RAGE,^[^ ^18^, ^19^ ^]^ and CCL2^[^ ^20^, ^21^ ^]^ have been verified to slow disease progression in animal models of progressive mesangioproliferative glomerulonephritis or type 2 diabetes. However, aptamers specifically designed for podocyte targeting have not yet been developed.

Therefore, in this study, we aimed to address this gap by developing an aptamer with specific affinity for injured podocytes. Using the Cell ‐ Systematic Evolution of Ligands by Exponential Enrichment (Cell‐SELEX) method, we screened podocytes subjected to various injuries, including adriamycin (ADR), puromycin aminonucleoside (PAN), and high glucose (HG) conditions. Through this approach, we successfully identified and optimized a novel aptamer that specifically binds to injured podocytes, both in vitro and in vivo, providing a novel strategy for targeted drug delivery in podocyte‐related kidney diseases.

## 2 Results

### 2.1 Aptamer Enrichment and Selection

**Figure 1:**
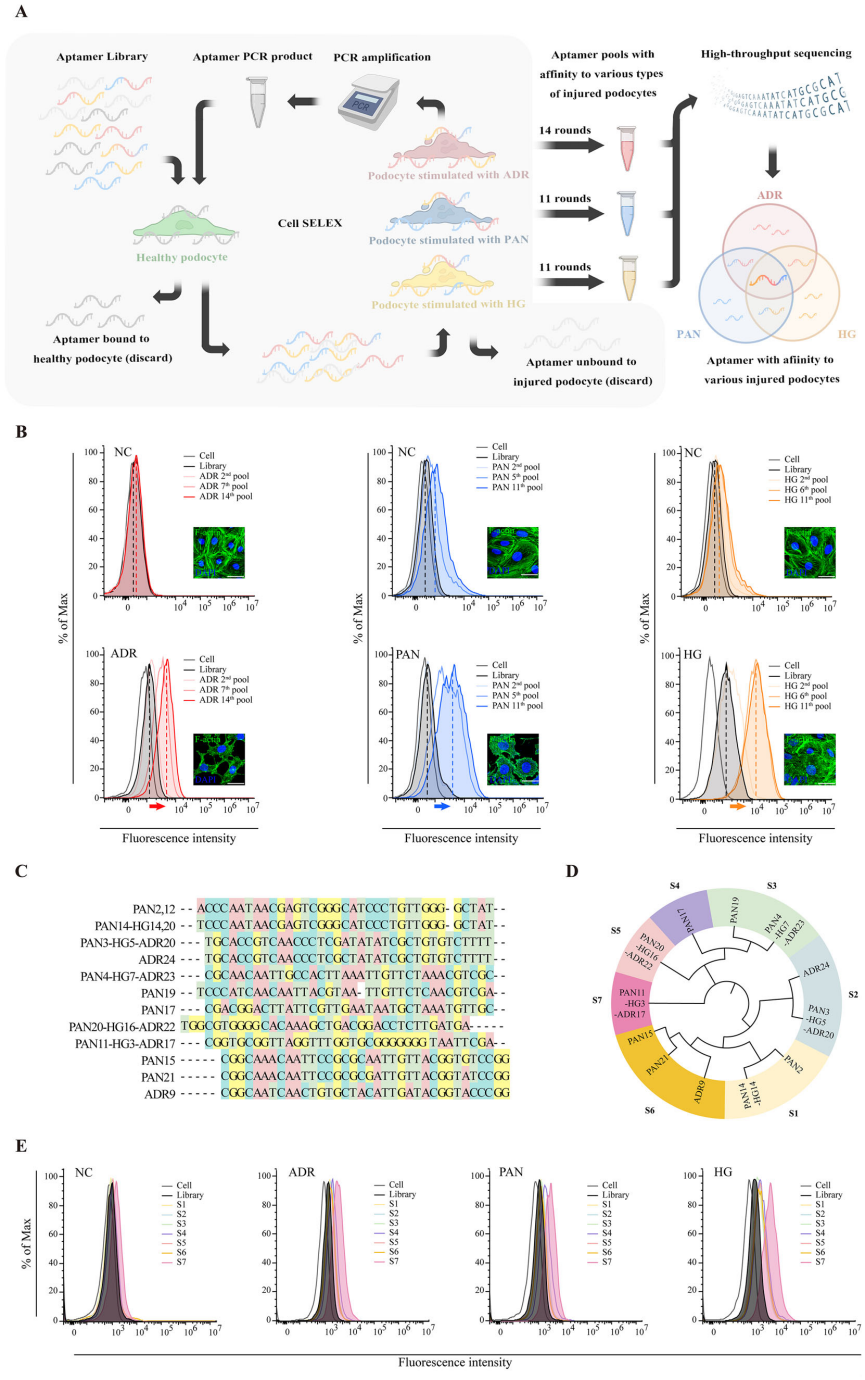
Identification and Characterization of Aptamers Targeting Injured Podocytes Using Cell‐SELEX. A) Schematic representation of the Cell‐SELEX process outlining the methodology for obtaining aptamers that specifically bind to injured podocytes. B) Analysis of the binding abilities of different aptamer pools to podocytes via flow cytometry. MPC5 cells were incubated with 250 nM of the amplified FAM‐labeled products at 4 °C. FAM‐labeled library was used as controls. Confocal images showing f‐actin in MPC5 cells after 48 h of treatment with 0.3 µg mL^−1^ adriamycin (ADR), 30 µg mL^−1^ puromycin aminonucleoside (PAN), or 50 mm high glucose medium (HG). Untreated MPC5 cells (NC) served as the control. Actin filaments were stained with FITC‐phalloidin (green), and nuclei were stained with DAPI (blue). Scale bars, 25 µm. C) Composition of the candidate aptamers, excluding the primer region. Candidate aptamers are named based on the type of injury, with numbers indicating their abundance rank. D) Phylogenetic tree showing the relationship among candidate aptamers. E) Flow cytometry analysis of the binding of seven candidate aptamers (S1‐S7).

The Cell‐SELEX process is outlined in **Figure** **1**A. The procedure began with the same initial single‐stranded DNA (ssDNA) oligo library, comprising 36 random nucleotides flanked by primer regions for PCR amplification. In the initial round, this library was incubated with normal podocytes to eliminate aptamers that bound nonspecifically. The remaining supernatant was then introduced to the injured podocytes to select aptamers with specific affinity. After washing, the aptamers that bound to the injured podocytes were harvested and amplified by PCR. The separated ssDNA pool from the PCR products was then applied to subsequent Cell‐SELEX cycles. The selection steps were repeated until aptamers with high affinity were enriched.

To initiate aptamer selection, we established three distinct podocyte injury models in MPC5 cells, using stimulations with ADR, PAN, or HG. All injury podocytes showed disrupted f‐actin skeletons in the podocytes compared with normal podocytes (Figure 1B). Flow cytometry was employed to monitor the screening process (Figure 1B). While the fluorescence intensity of the 2nd pool was almost equivalent to that of the initial library for injured podocytes, the pools after 5–7 rounds of selection showed a significant increase in fluorescence intensity compared to the initial library. In contrast, for normal podocytes used as the control, only a minor increase in fluorescence intensity was observed. These findings suggest that the ssDNA pools with an affinity for the target cells were effectively enriched. In addition, minimal difference in fluorescence was observed between the 11th–14th pool, suggesting that further screenings might not yield a ssDNA pool with improved binding.

Therefore, we subjected ssDNA pools from the three rounds of Cell‐SELEX to high‐throughput sequencing. A total of 23848 sequences were detected with a total of 15 million occurrences. These sequences were then sorted by abundance in descending order and renamed according to their grouping and ranking. We primarily focused on the top 100 sequences, as they represented the majority of aptamers in the final pool (the pool with the highest binding affinity). We compared their abundance between the final and the 2nd pool, preserving those sequences that showed an increase in abundance and discarding those without significant changes or with decreased abundance. Next, priority was given to sequences found in multiple injury models, as we aimed to identify aptamers with affinity for various types of injured podocytes. This finally led to the selection of 12 candidate sequences (Figure 1C). Phylogenetic tree analysis, conducted with MEGA 11, categorized these into 7 major groups and showed their relationship in composition (Figure 1D).

Subsequently, 7 corresponding oligonucleotides were synthesized as candidate aptamers (Table S1, Supporting Information), and their binding affinity to podocytes was assessed using flow cytometry. Among the candidate sequences, aptamer S7 demonstrated the highest binding affinity to injured podocytes (Figure 1E).

### 2.2 Optimization and Characterization of Aptamer

**Figure 2:**
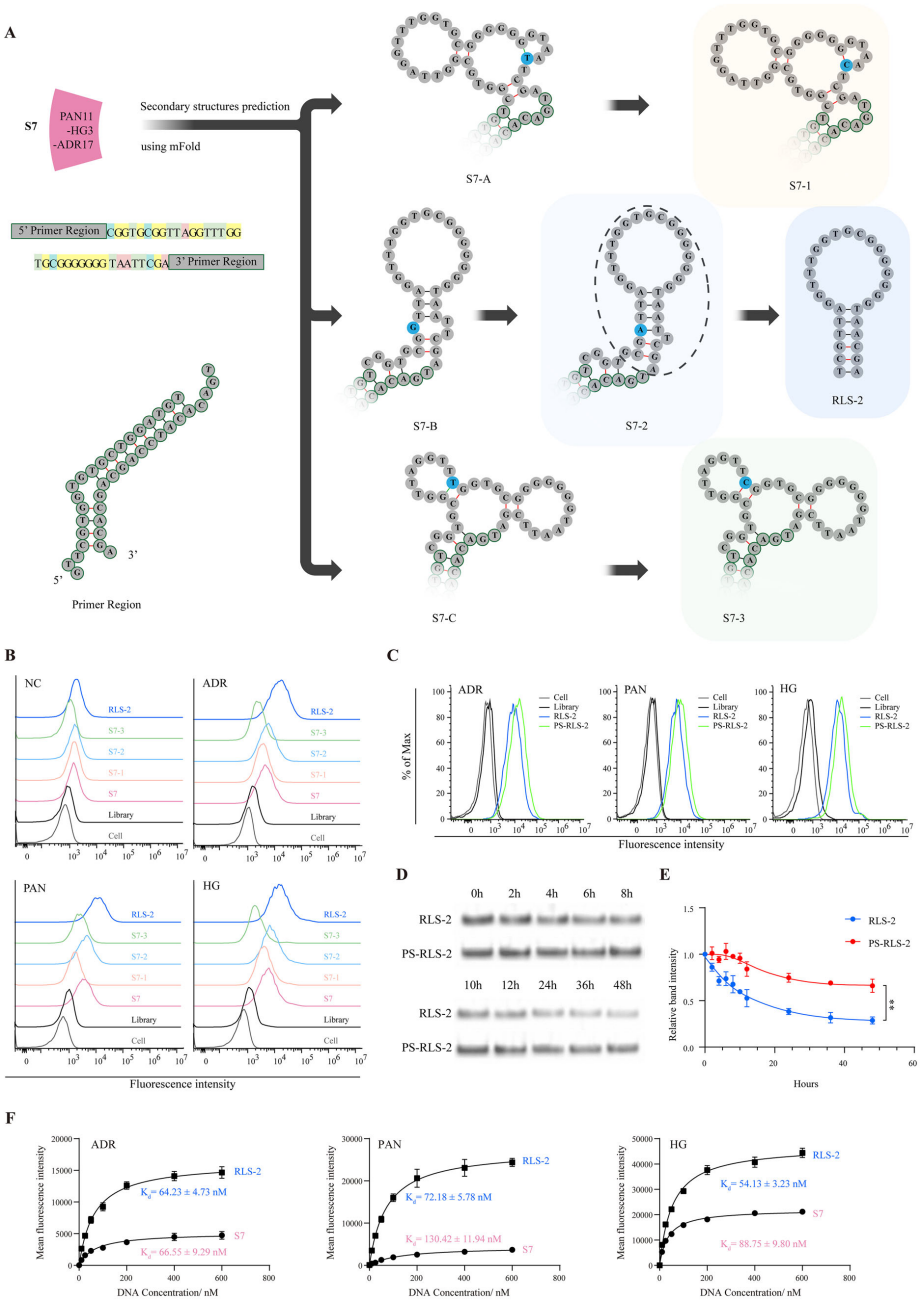
Optimization and Characterization of S7 Variants and RLS‐2 for Podocyte Targeting. A) Predicted secondary structures of S7 (S7‐A, S7‐B, S7‐C) and its derived variants (S7‐1, S7‐2, S7‐3). The primer region, indicated by nucleotides with green borders, is shown separately. Mismatched nucleotides in S7 (highlighted in blue) were modified to create the variants. RLS‐2 was derived from S7‐2 by truncation, as denoted by the dashed circle. B) Binding ability of S7, its variants, and RLS‐2 to podocytes, as determined by flow cytometry. C) Binding ability of RLS‐2 and its phosphorothioate‐modified variant (PS‐RLS‐2) to injured podocytes, as analyzed by flow cytometry. D‐E) Evaluation of the serum stability of RLS‐2 and PS‐RLS‐2 in a 10% serum incubation medium (D), and the corresponding quantitative analysis (E) with data presented as mean ± standard deviation. *n* = 3. ^**^ *p* < 0.01. F) The dissociation constant (K_d_) curves and statistical analysis for S7 and RLS‐2, with data presented as mean ± standard deviation. *n* = 3.

To enhance its binding properties, we optimized S7 based on its predicted secondary structures (**Figure** **2**A). The prediction results of mfold outlined three main secondary structures for S7 (S7‐A, S7‐B, S7‐C), each containing mismatched nucleotides. To determine which structure contributed most to binding, we created three variants (S7‐1, S7‐2, S7‐3) by rectifying the mismatches. Additionally, to lower synthesis costs and enhance the internalization of the aptamers, we designed the four S7‐2 truncated form named RLS‐1 to RLS‐4. The composition of all these aptamers is detailed in Table S2 (Supporting Information).

**Figure 3:**
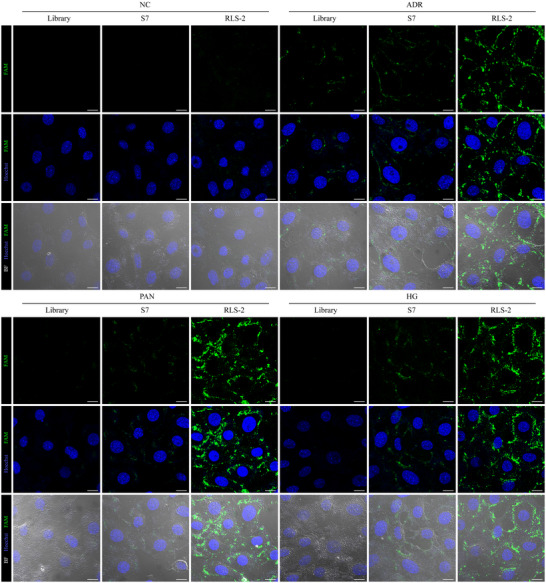
Binding of RLS‐2 to Injured Podocytes Analyzed by Confocal Microscopy. Binding ability of RLS‐2 to injured podocytes analyzed by confocal microscopy. Hoechst 33 342 staining (blue) indicates cell nuclei, while RLS‐2 is represented by green signals. Scale bars, 10 µm.

The flow cytometry results indicated that S7‐2 was the only variant that maintained the binding ability of S7 (Figure 2B). Among the four truncated variants of S7‐2 (RLS‐1 to RLS‐4), RLS‐2 exhibited the strongest binding ability at 37 °C (Figure S1, Supporting Information), with only a slight decrease compared to the binding at 4 °C (Figure S2, Supporting Information). Flow cytometry analysis (Figure 2B) and confocal microscopy (**Figure** **3**) confirmed that RLS‐2 exhibited stronger binding to injured podocytes compared to the original S7. On the contrary, a slight increase in fluorescence intensity was observed in normal podocytes or when incubated with the control aptamer. Confocal microscopy images further highlighted the differences in binding between S7 and RLS‐2. While both S7 and RLS‐2 showed visible fluorescence in injured podocytes, RLS‐2 exhibited significantly stronger fluorescence. In contrast, the binding fluorescence of RLS‐2 to normal podocytes was weak. The images also indicated that the bound aptamers were primarily located on the cell surface. As a control, the initial library showed hardly any binding to healthy podocytes.

To further evaluate the binding affinity of the aptamers to injured podocytes, we determined their dissociation constant (K_d_) values (Figure 2F). The K_d_ values for S7 in injured podocytes were measured at 66.55 ± 9.29 nM (ADR), 88.75 ± 9.80 nM (PAN), and 130.42 ± 11.94 nM (HG). For RLS‐2, the corresponding K_d_ values were 64.23 ± 4.73 nM (ADR), 54.13 ± 3.23 nM (PAN), and 72.18 ± 5.78 nM (HG). These results demonstrate that RLS‐2, the optimized aptamer, exhibited lower K_d_ values and higher affinity for injured podocytes, indicating our successful optimization.

Enhancing aptamer resistance to nucleases at 37 °C is crucial for in vivo applications. Thus, we modified RLS‐2 with three methods: phosphorothioate, 2'‐fluoro (2′‐F), and 2′‐O‐methyl (2′‐O‐Me modifications) (Table S3, Supporting Information). The results showed that the phosphorothioate modification had little effect on the binding ability of RLS‐2 (Figure 2C) and enhanced the stability of RLS‐2 (Figure 2D,E), while the other two modifications reduced its binding (Figure S3, Supporting Information).

Competitive binding assays demonstrated that RLS‐2 could effectively compete with S7 for the same binding sites on injured podocytes (Figure S4, Supporting Information), and treatment with proteinase further confirmed that RLS‐2 binds to protein targets on the podocyte surface (Figure S5, Supporting Information).

### 2.3 The Binding of RLS‐2 in Human Kidney Cell Lines

**Figure 4:**
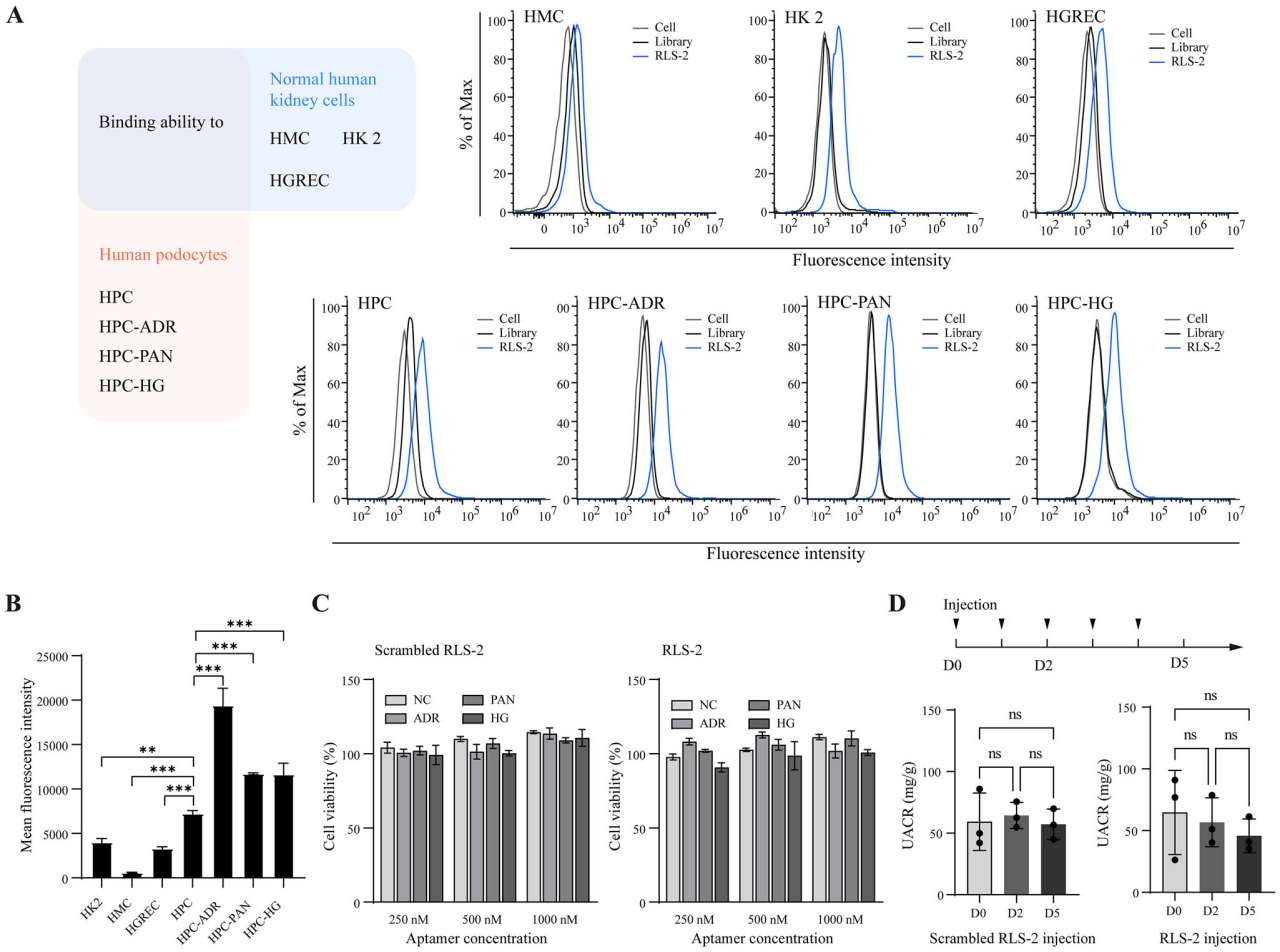
Characterization of RLS‐2: Binding Efficiency, Serum Stability, and Safety in Kidney Cell Lines. A) Binding assays of RLS‐2 with various human‐derived kidney cell lines, including human mesangial cells (HMC), human proximal tubular epithelial cells (HK‐2), human renal glomerular endothelial cells (HGREC), and human podocytes (HPC). HPC‐ADR, HPC‐PAN, and HPC‐HG represent injured human podocytes induced by different methods. B) Quantification of RLS‐2‐specific binding after subtraction of library fluorescence intensity. Error bars indicate mean ± standard deviation. *n* = 3. ^**^ *p* < 0.01; ^***^ *p* < 0.001. C) Cell viability of podocytes after 24 h treatment with scrambled RLS‐2 and RLS‐2, evaluated using the CCK‐8 assay. Error bars indicate mean ± standard deviation. D) Urinary albumin‐to‐creatinine ratio (UACR) in BALB/c mice following five consecutive days of aptamer administration (0.16 nmol g^−1^ body weight per day) via tail vein injection. Error bars indicate mean ± standard deviation. *n* = 3. ns, not significant.

In addition to demonstrating the binding in mouse‐derived podocyte cell line MPC5, we also tested the binding of RLS‐2 in human kidney cell lines including human proximal tubular epithelial cells (HK‐2), human podocytes (HPC), human mesangial cells (HMC), and human renal glomerular endothelial cells (HRGECs). For HPC, injury models were induced using the same methods as those applied to mouse podocytes, resulting in HPC‐ADR, HPC‐PAN, and HPC‐HG models. As shown in **Figure** **4**A,B, among the normal cultured HK‐2, HMC, HRGEC, and HPC, the HPC showed the highest fluorescence intensity following incubation with RLS‐2. Additionally, all injured HPC models exhibited higher fluorescence intensity compared to normal HPC. These findings suggest that RLS‐2 binds more strongly to injured human podocytes compared to normal podocytes or other types of human‐derived kidney cell lines.

### 2.4 The Cytotoxicity of RLS‐2

Considering the desire to use RLS‐2 as a targeting drug carrier in the future, we explored whether RLS‐2 has any cytotoxic effects on podocytes. For the control, the RLS‐2 sequence was scrambled (Table S2, Supporting Information). We carried out a cytotoxicity assay on the MPC5 cell line, treating the cells with various concentrations of scrambled RLS‐2 and RLS‐2. According to the results, both scrambled RLS‐2 and RLS‐2 did not impact the viability of either normal or injured podocytes after 24 h of incubation at concentrations of 25, 500, or 1000 nM (Figure 4C). In addition, no significant increase in the albumin‐to‐creatinine ratio (UACR) was observed in urine from healthy mice after continuous injection of either scrambled RLS‐2 or RLS‐2 for 5 days (Figure 4D). These results indicate that RLS‐2 is safe for future applications.

### 2.5 Cellular Internalization of RLS‐2

**Figure 5:**
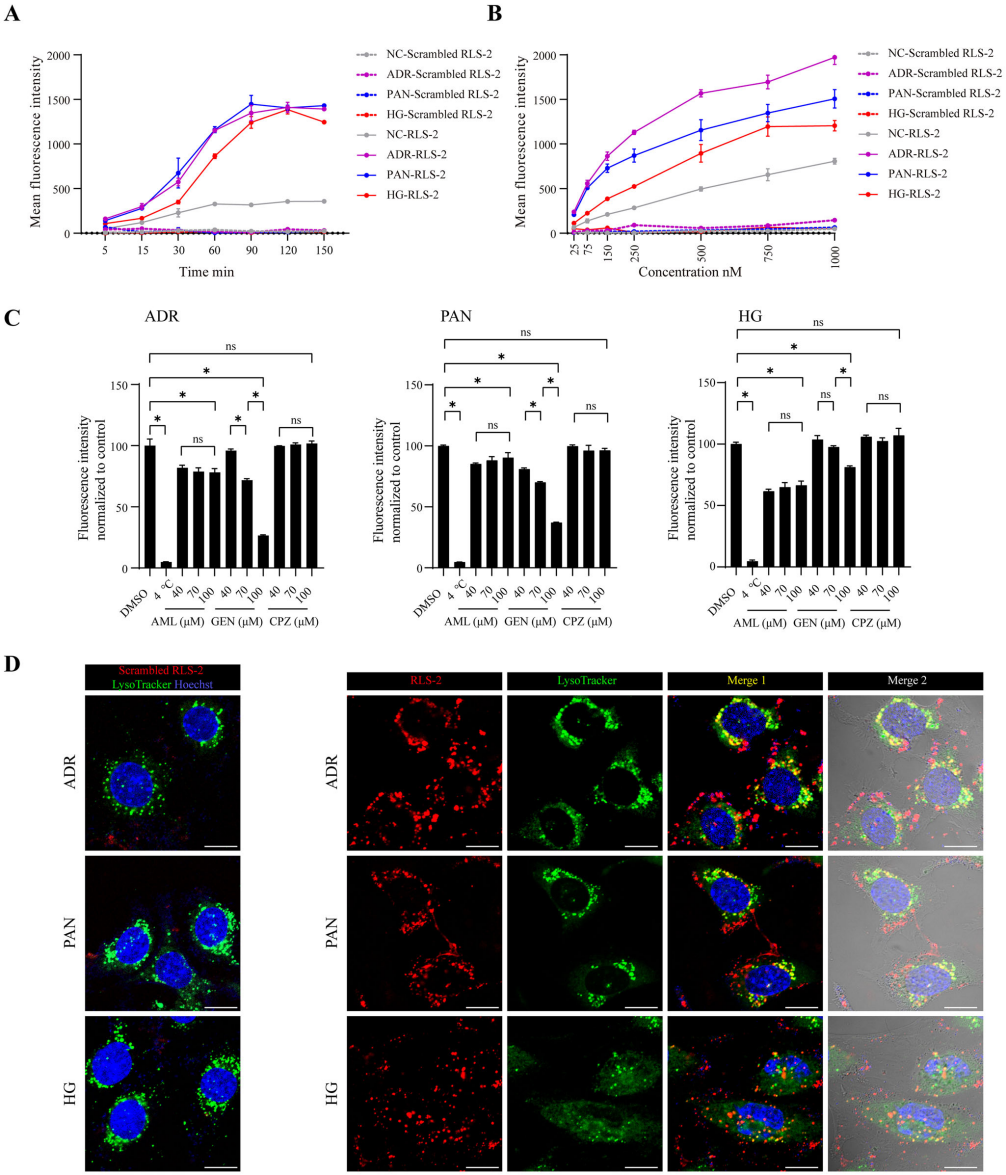
Cellular Uptake and Intracellular Trafficking of RLS‐2 in Podocytes: Effects of Time, Concentration, and Endocytic Pathways. A,B) Time‐dependent (A) and concentration‐dependent (B) cellular uptake of scrambled RLS‐2 and RLS‐2 in podocytes analyzed by flow cytometry. Data are presented as mean ± standard deviation. *n* = 3. C) Percentage of RLS‐2 uptake by injured podocytes at 4 and 37 °C following a 30 min pre‐treatment with endocytosis inhibitors (AML: amiloride, GEN: genistein, CPZ: chlorpromazine). Uptake in cells treated with DMSO was considered 100%. Error bars indicate mean ± standard deviation. *n* = 3. ^*^, *p* < 0.05; ns, not significant. D) Representative confocal microscopy images showing the distribution of Cy5‐labeled aptamers (red) and their co‐localization with a lysosome marker (LysoTracker Green DND‐26, green) in injured MPC5 cells after a 60‐min incubation at 37 °C. Nuclei were counterstained with Hoechst 33 342 (blue). Scale bars, 10 µm.

To determine whether injured podocytes could internalize RLS‐2 in vitro, we incubated these cells with FAM‐labeled RLS‐2 at 37 °C. The cellular uptake was then assessed using flow cytometry, after removing surface‐bound aptamers with trypsin‐EDTA. RLS‐2 was significantly accumulated in injured podocytes, whereas the control sequence, scrambled RLS‐2, showed minimal internalization (**Figure** **5**A). The uptake of RLS‐2 reached equilibrium after 90 min of incubation, with fluorescence intensity reaching a plateau. Interestingly, healthy podocytes were also capable of uptaking RLS‐2, although to a lesser extent compared to injured podocytes. Furthermore, we evaluated the effect of aptamer concentrations on cellular uptake (Figure 5B). It revealed that the accumulation of RLS‐2 in podocytes increased with aptamer concentrations ranging from 25 to 1000 nM. In summary, RLS‐2 was internalized by injured podocytes in both a time‐dependent and concentration‐dependent manner.

Next, we investigated the endocytic pathway involved in RLS‐2 uptake (Figure 5C). The accumulation of RLS‐2 in injured podocytes was completely halted at 4 °C, suggesting that the internalization of RLS‐2 is an energy‐dependent process. We further examined the cellular uptake in injured podocytes pre‐treated with various endocytosis inhibitors: amiloride, a macropinocytosis inhibitor; genistein, an inhibitor of the caveolae‐dependent pathway; and chlorpromazine, a clathrin‐dependent pathway inhibitor. The results showed that the uptake was predominantly hindered by genistein, with a slight inhibition observed with amiloride in the ADR and PAN groups. In the HG model, both amiloride and genistein demonstrated similar inhibitory effects. Chlorpromazine did not affect endocytosis in any of the groups. These findings imply that RLS‐2 internalization in injured podocytes occurs primarily via the caveolae‐dependent pathway and macropinocytosis.

Following the determination of the endocytic pathway, we further examined the intracellular distribution of RLS‐2. Injured podocytes were incubated with Cy5‐labeled aptamers and FITC‐labeled organelle markers. In line with the results of cellular uptake, confocal laser microscopy images revealed a significant accumulation of RLS‐2 within injured podocytes, characterized by a punctate pattern around the nucleus. Conversely, only a minimal presence of scrambled RLS‐2 was observed inside the cells. Additionally, there was noticeable co‐localization of RLS‐2 with lysosomes, while there was no obvious overlap with the endoplasmic reticulum (Figure 5D; Figure S6, Supporting Information). These observations suggest that most RLS‐2 were transported to lysosomes after internalization. Interestingly, a small portion of RLS‐2 near the nucleus did not co‐localize with lysosomes, indicating that some aptamers may escape lysosomal degradation.

### 2.6 The Biodistribution of the Aptamer In Vivo

**Figure 6:**
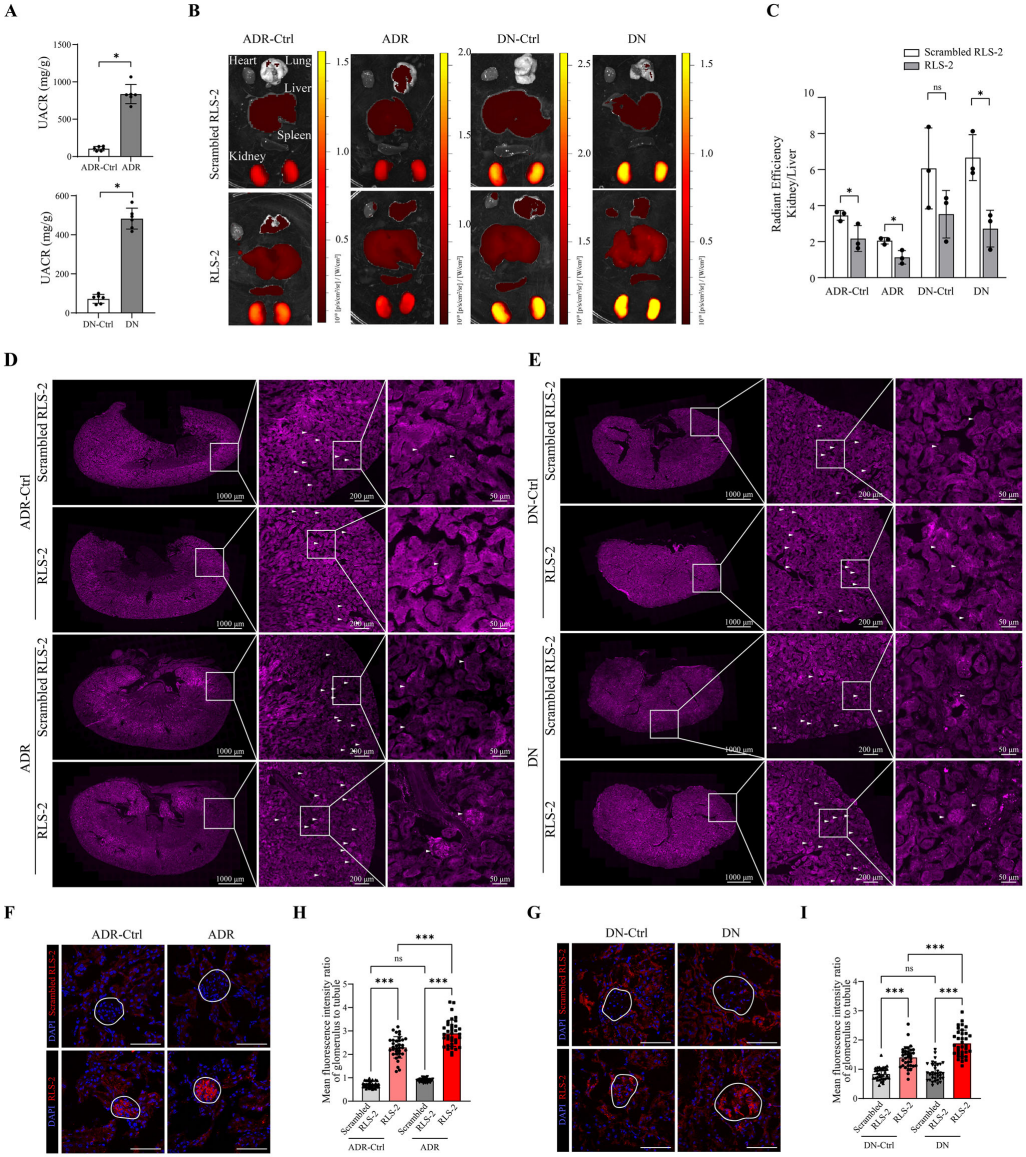
Aptamer Distribution in Mouse Models of Nephropathy. A) UACR for ADR and DN mice. *n* = 6. ^*^ *p* < 0.05. B) Luminescence imaging of various organs (heart, lungs, liver, spleen, and kidneys) from mice, taken 1 h after Cy5‐labeled aptamer injection. Scrambled RLS‐2 was used as the control aptamer. C) Quantitative analysis of the kidney‐to‐liver ratio in mice. Error bars indicate mean ± standard deviation. *n* = 3. ^*^ *p* < 0.05; ns, not significant. D,E) Distribution of scrambled RLS‐2 and RLS‐2 in different mouse groups. From left to right, images show zoomed‐in views of the regions marked by white boxes. White arrows indicate the glomerular region. F,G) Representative confocal microscopy images showing the distribution of aptamers in glomeruli (indicated by white circles). Nuclei were stained with DAPI (blue). Scale bar, 50 µm. H,I) Quantitative analysis of the mean fluorescence intensity ratio of glomerulus to tubule. Error bars indicate mean ± standard deviation. *n* = 30. ^***^ *p* < 0.001; ns, not significant. Group designations: ADR‐Ctrl (healthy BALB/c), ADR (adriamycin‐induced nephropathy in BALB/c mice), DN‐Ctrl (healthy DBA/2), and DN (STZ‐induced diabetic nephropathy in DBA/2 mice).

Having characterized properties of RLS‐2 in vitro, we further investigated its binding ability in mouse models. To align with the cell models previously used, we induced ADR nephropathy in BALB/c mice and diabetic nephropathy in DBA/2 mice using streptozotocin (STZ). We did not use the PAN nephropathy model, as it is more commonly applied to rats rather than mice.^[^ ^22^ ^]^ Both ADR and diabetic nephropathy mouse models exhibited weight loss and an increased kidney weight to body weight ratio (Table S4, Supporting Information). The blood glucose level in diabetic mice eventually reached 26.07 ± 1.80 mm (Table S4, Supporting Information). Additionally, the UACR significantly increased in each nephropathy group compared to the control group (**Figure** **6**A). Renal histomorphological examination confirmed glomerular lesions in both models (Figure S7, Supporting Information). The ADR nephropathy mice displayed podocyte vacuolar degeneration and protein casts, whereas the diabetic nephropathy mice showed mesangial cell proliferation and increased mesangial matrix. Both groups exhibited glomerular fibrosis and reduced podocin expression. These findings confirm the successful establishment of the ADR and diabetic nephropathy mouse models.

To ascertain the in vivo biodistribution of RLS‐2, we administered 0.16 nmol g^−1^ body weight of Cy5‐labeled aptamers to mice via tail vein injection. One hour after injection, the mice were euthanized, and major organs including the heart, lungs, liver, spleen, and kidneys were collected. The fluorescence images captured by the live animal imaging system revealed that RLS‐2 and scrambled RLS‐2 predominantly accumulated in the kidneys in all groups (Figure 6B). The liver and spleen exhibited secondary distribution, whereas the heart and lungs displayed only weak fluorescence. We calculated the kidney‐to‐liver ratios based on radiant efficiency to evaluate renal targeting, which showed ratios in RLS‐2 injection groups were lower than scrambled RLS‐2 (Figure 6C). Furthermore, we characterized the distribution of RLS‐2 in liver and spleen tissues using fluorescence microscopy (Figure S8, Supporting Information). The results showed that RLS‐2 was predominantly distributed in liver cells, exhibiting a diffuse pattern. In spleen tissue, RLS‐2 was mainly located in areas outside the lymphatic follicles, showing a scattered, punctate distribution.

We further examined the renal distribution of the aptamers at the histological level (Figure 6D,E). Both RLS‐2 and scrambled RLS‐2 were present in the tubular area. By staining for the proximal tubular marker AQP1, we found that aptamers were primarily distributed within tubular cells, rather than in the filtrate (Figure S9, Supporting Information). In addition, RLS‐2 accumulated more significantly in the glomerular area, while scrambled RLS‐2 showed an almost negligible deposition in the glomerular area. In nephropathy mice, the glomerular fluorescence of RLS‐2 was higher than in the control group. This suggests that RLS‐2 specifically binds to glomeruli in nephropathy mice. To quantify these differences, we assessed the mean fluorescence intensity ratio of glomerulus to tubule from 30 fluorescence images per group (Figure 6F–I). The ratio in the RLS‐2 injected group was almost threefold higher in diabetic nephropathy mice and fourfold higher in ADR nephropathy mice compared to the scrambled RLS‐2 group. Conversely, scrambled RLS‐2 showed no significant difference between nephropathy and control groups. These results demonstrate that RLS‐2 is highly specific to glomeruli in vivo, particularly in the glomeruli of nephropathy mice. In addition, we interestingly observed strong nuclear binding of RLS‐2 in frozen kidney sections from human and mouse (Figure S10A,B, Supporting Information). Specifically, RLS‐2 injected to mice was distributed in the glomerular region in vivo, while in the staining of frozen sections, it predominantly localized to the nuclear region (Figure S10B, Supporting Information).

**Figure 7:**
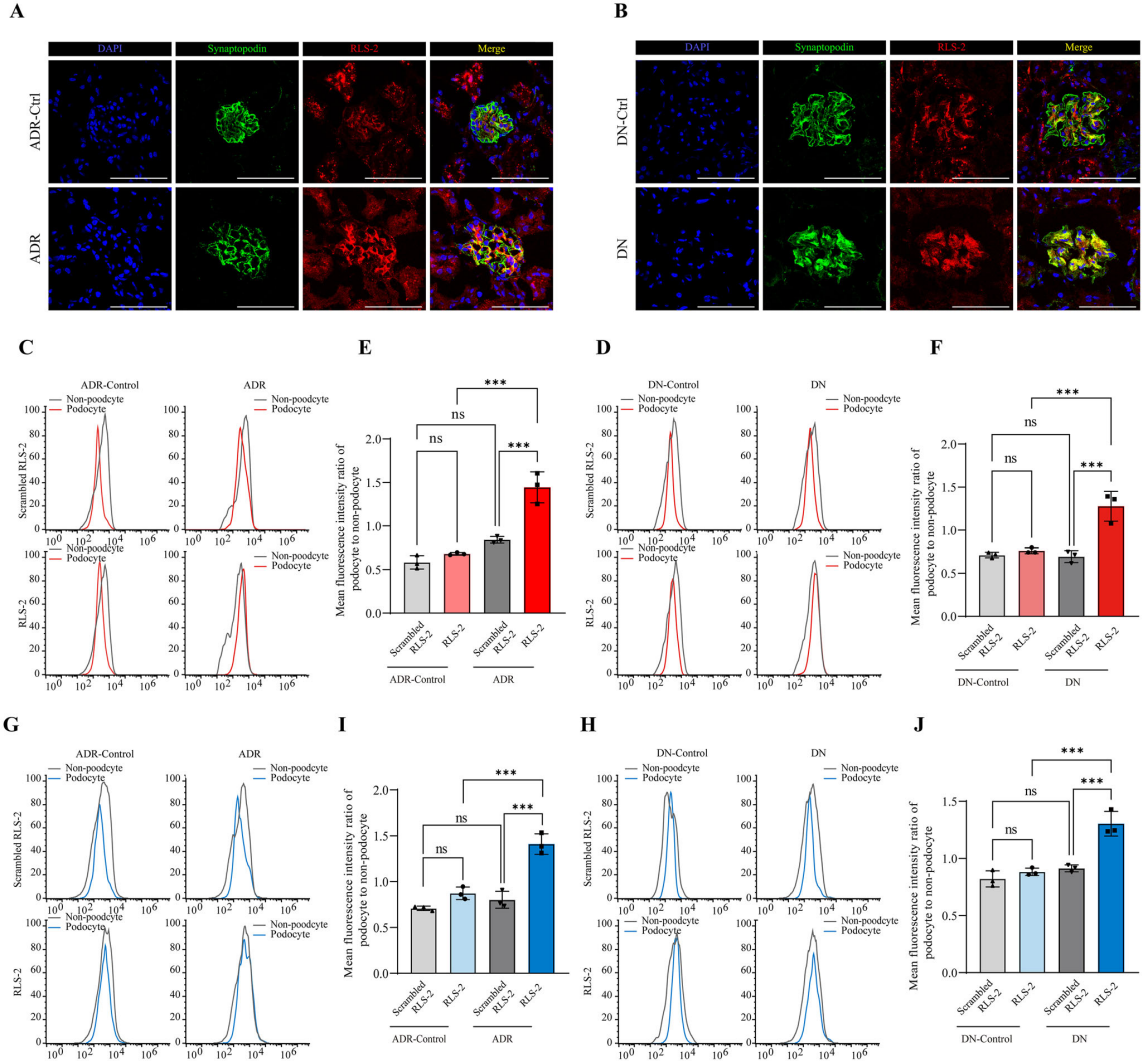
Verification of RLS‐2 Distribution in Podocytes. A,B) Representative confocal images showing the co‐localization of RLS‐2 (red) with the podocyte marker synaptopodin (green). Nuclei were stained with DAPI (blue). Scale bars, 50 µm. C,D) Representative flow cytometry results showing the mean fluorescence intensity in podocytes and non‐podocytes from mice after tail vein injection of FAM‐labeled aptamer. E–F). Quantitative analysis of the mean fluorescence intensity ratio of podocyte to non‐podocyte. Error bars indicate mean ± standard deviation. *n* = 3. ^***^ *p* < 0.001; ns, not significant. G,H) Representative flow cytometry results showing the mean fluorescence intensity in podocyte and non‐podocyte from kidney cell suspensions of different mouse groups after in vitro incubation with the FAM‐labeled aptamer. I,J) Quantitative analysis of the mean fluorescence intensity ratio of podocyte to non‐podocyte. Error bars indicate mean ± standard deviation. *n* = 3. ^***^ *p* < 0.001; ns, not significant.

Lastly, we investigated whether RLS‐2′s binding site in the glomerulus is the podocyte. Confocal microscopy images revealed significant co‐localization of RLS‐2 with synaptopodin, a podocyte‐specific marker protein (**Figure** **7**A,B). We also used flow cytometry to validate the in vivo distribution of RLS‐2. After intravenous injection of the aptamers, mouse kidneys were digested into cell suspensions. The results showed that nephropathy mice injected with RLS‐2 had a significantly higher fluorescence intensity ratio of podocyte to non‐podocyte than other groups (Figure 7C–F). The ratio in nephropathy mice with RLS‐2 injection was 1.44 ± 0.18 (ADR) and 1.28 ± 0.17 (DN), respectively, while the ratio in mice with scrambled RLS‐2 injection was below 1 (ranging from 0.58 to 0.84), demonstrating the specific distribution of RLS‐2 to injured podocytes in vivo. Additionally, we co‐incubated renal cell suspensions with the aptamer in vitro to further confirm the binding of RLS‐2 to podocytes (Figure 7G–J). These results indicate that RLS‐2 binds to podocytes within glomeruli in vitro and in vivo.

### 2.7 Target Protein Identification

**Figure 8:**
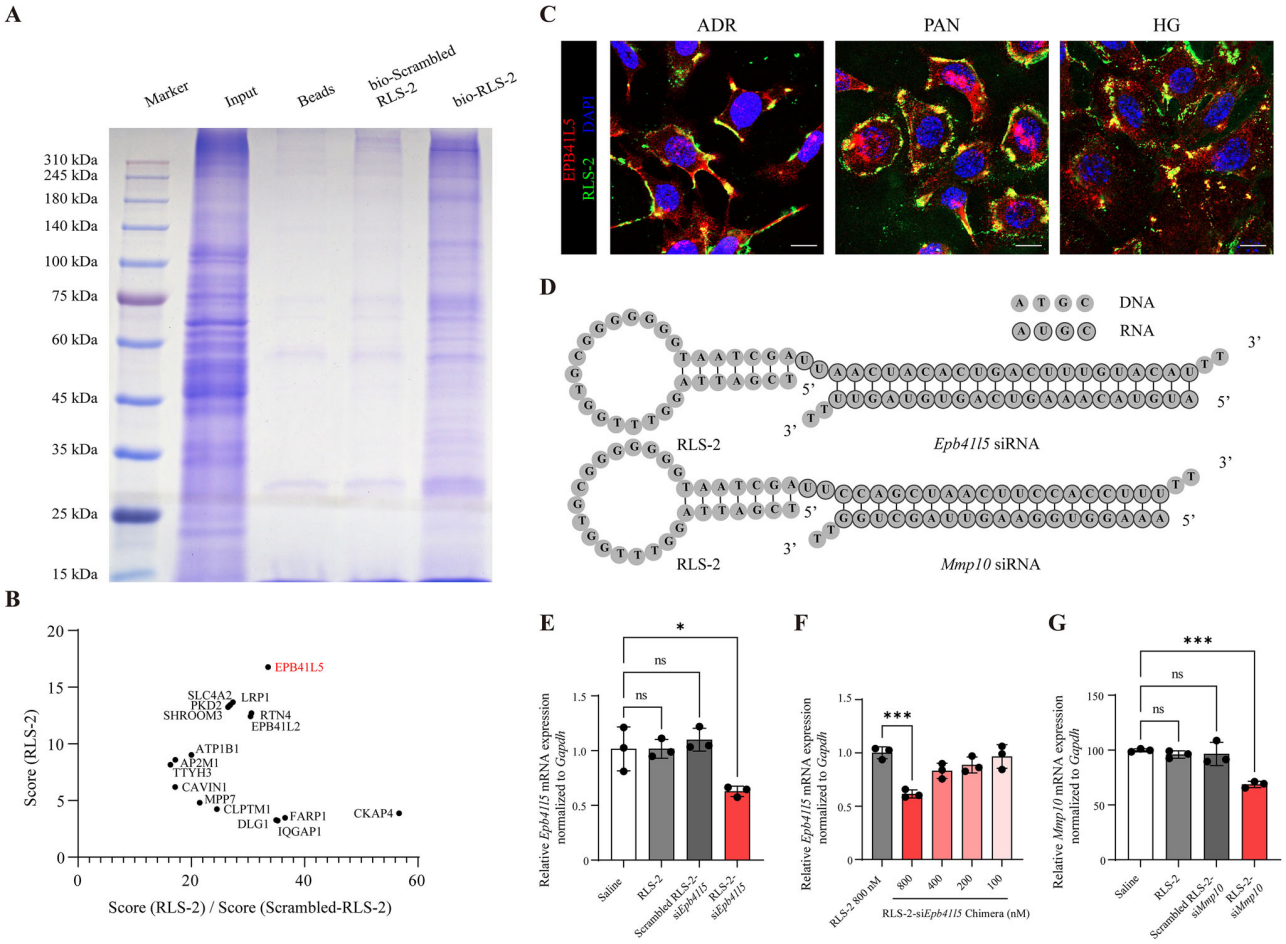
Identification of RLS‐2 Target Protein and Knockdown efficiency of RLS‐2–siRNA Chimeras. A) Coomassie Brilliant Blue staining was used to analyze proteins separated by SDS‐PAGE, including input protein (Input), beads pull‐down protein (Beads), biotin‐conjugated scrambled RLS‐2 pull‐down protein (bio‐Scrambled RLS‐2), and biotin‐conjugated RLS‐2 pull‐down protein (bio‐RLS‐2). B) The spectrum score of the candidate proteins in the RLS‐2 pulldown group (Score (RLS‐2)) and the ratio of spectrum score (RLS‐2) to spectrum score (Scrambled‐RLS‐2). C) Representative images showing the co‐localization of RLS‐2 (green) and EPB41L5 (red). Nuclei were stained with DAPI (blue). Scale bars, 10 µm. D) Schematic representation of RLS‐2‐si*Epb41l5* and RLS‐2‐si*Mmp10* chimeras. E) Quantitative analysis of qRT‐PCR results showing the knockdown efficiency of the RLS‐2‐si*Epb41l5* chimera (800 nM) in normal MPC5 cells after 24 h incubation. Saline, RLS‐2 (800 nM), and scrambled RLS‐2‐si*Epb41l5* chimera (800 nM) were used as controls. Error bars indicate mean ± standard deviation. *n* = 3. ^*^ *p* < 0.05; ns, not significant. F) Quantitative analysis of qRT‐PCR results showing the knockdown efficiency at different concentrations of the RLS‐2‐si*Epb41l5* chimera. 800 nM RLS was used as the control. Error bars indicate mean ± standard deviation. *n* = 3. ^***^ *p* < 0.001. G) Quantitative analysis of qRT‐PCR results showing the knockdown efficiency of the RLS‐2‐si*Mmp10* chimera (800 nM) in normal MPC5 cells. Saline, RLS‐2 (800 nM), and scrambled RLS‐2‐si*Mmp10* chimera (800 nM) were used as controls. Error bars indicate mean ± standard deviation. *n* = 3. ^***^ *p* < 0.001; ns, not significant.

To further investigate the potential targets of RLS‐2, biotin‐conjugated aptamers (bio‐Scrambled RLS‐2, bio‐RLS‐2, shown in Table S3, Supporting Information) were synthesized for a pulldown assay. Prior to the experiment, it was confirmed that the biotin modification did not affect the binding ability of RLS‐2 (Figure S11, Supporting Information). Total membrane protein fractions from high‐glucose‐damaged podocytes were extracted and incubated with biotin‐conjugated RLS‐2, scrambled RLS‐2, or a non‐aptamer buffer. Streptavidin‐agarose beads were used to precipitate the aptamer‐protein complexes. Protein bands were visualized using SDS‐PAGE followed by Coomassie Brilliant Blue staining. The results showed multiple protein bands in the bio‐RLS‐2 lane, while bio‐Scrambled RLS‐2 lane showed significantly fewer bands (**Figure** **8**A). To identify the differential proteins, the bio‐Scrambled RLS‐2 and bio‐RLS‐2 lanes were excised and sent for LC‐MS/MS analysis. For the mass spectrometry results analysis, cytoplasmic proteins, nuclear proteins, and organelle proteins (e.g., mitochondrial and ribosomal proteins) were excluded, because they are unlikely to directly interact with the aptamer. Since RLS‐2 specifically bound to damaged podocytes, proteins with higher mass spectrometry score ratios (RLS‐2/Scrambled‐RLS‐2) were retained. In the end, 17 candidate proteins were identified (Table S5, Supporting Information). Among these, Band 4.1‐like protein 5 (EPB41L5) showed both a high mass spectrometry score (RLS‐2) and a high score ratio (RLS‐2/Scrambled‐RLS‐2) (Figure 8B), and its MS/MS spectrum is shown in Figure S12 (Supporting Information). Furthermore, confocal microscopy revealed co‐localization of RLS‐2 and EPB41L5 on the cell membranes of three different injured podocytes (Figure 8C), indicating that EPB41L5 is a target protein of RLS‐2.

### 2.8 siRNA Delivery via AsiCs

To investigate the ability of RLS‐2 to deliver therapeutic nucleic acids into podocytes, we constructed aptamer‐siRNA chimeras (AsiCs). As a first step, we attempted to deliver siRNA targeting *Epb41l5*. RLS‐2 was linked to the siRNA sense strand via a “UU” linker, with “TT” overhangs added to both the sense and antisense strands to enhance stability (Figure 8D; Table S6, Supporting Information). AsiCs were assembled through gradient cooling in a PCR thermocycler. The results showed that RLS‐2‐si*Epb41l5* downregulated *Epb41l5* expression in podocytes by ≈ 40%, whereas scrambled RLS‐2‐si*Epb41l5* had no such effect (Figure 8E). Furthermore, the inhibitory effect of RLS‐2‐si*Epb41l5* on gene expression was dose‐dependent (Figure 8F). To assess whether RLS‐2 could serve as a general siRNA carrier, we further conjugated it with siRNA targeting *Mmp10*. The results demonstrated that RLS‐2‐si*Mmp10* reduced *Mmp10* expression in podocytes by ≈30% (Figure 8G).

## 3 Discussion

Many current treatments for kidney diseases are associated with a range of adverse effects. Targeted drug delivery has emerged as a promising approach, offering enhanced efficacy, reduced side effects, and the potential to facilitate gene therapies. Significant advancements in renal targeting have led to improvements in drug utilization efficiency^[^ ^23^, ^24^, ^25^ ^]^ and gene expression modulation,^[^ ^26^, ^27^, ^28^ ^]^ highlighting the transformative potential of this approach. However, research specifically focusing on podocyte‐targeted delivery remains limited. Antibodies have been used in some studies for podocyte targeting.^[^ ^10^, ^29^ ^]^ Given the correlations between antibodies and glomerular injury^[^ ^30^ ^]^ and idiopathic nephrotic syndrome,^[^ ^31^, ^32^, ^33^, ^34^ ^]^ safety is the biggest concern. In this research, we have successfully identified and optimized a specific aptamer, RLS‐2, a novel aptamer that selectively targets injured podocytes in both in vivo and in vitro. Compared to existing renal targeting methods such as viral vectors,^[^ ^35^ ^]^ kidney targeting peptide,^[^ ^36^ ^]^ and extracellular vesicles,^[^ ^37^ ^]^ RLS‐2 offers distinct advantages, including high affinity, low immunogenicity, and low synthesis costs. Additionally, the versatility of chemical modifications allows RLS‐2 to enhance performance^[^ ^38^ ^]^ or integrate with various drug carriers,^[^ ^39^, ^40^ ^]^ positioning it as a highly competitive candidate for podocyte‐targeted therapy.

RLS‐2 was derived from S7 through optimization and truncation based on its predicted secondary structure. RLS‐2 exhibited a lower dissociation constant and higher maximum binding capacity, indicating enhanced affinity for injured podocytes. These attributes are crucial for improving the delivery of therapeutic payloads to podocytes, which are essential targets in nephropathy.

Additionally, RLS‐2 retained its binding affinity at physiological temperatures and exhibited no significant cytotoxicity to podocytes, making it suitable for potential in vivo applications. 2′‐OMe,^[^ ^41^ ^]^ 2′‐F^[^ ^42^ ^]^ and phosphorothioate^[^ ^43^ ^]^ modifications are generally used to improve stability of aptamers. However, both 2′‐OMe‐ and 2′‐F‐modified RLS‐2 showed lower binding capacity compared to the phosphorothioate‐modified RLS‐2. Therefore, to enhance resistance of RLS‐2 to nucleases, phosphorothioate modification was employed, which improved its stability in vitro without significant impact on its binding ability.

Access to targeted cells is a critical initial step in drug delivery, but the therapeutic efficacy of most drugs also depends on their ability to enter cells.^[^ ^44^ ^]^ Our in vitro cellular internalization experiments demonstrated RLS‐2′s efficient entry into injured podocytes in both time‐dependent and concentration‐dependent manners, which is vital for drug delivery. Additionally, since the mean fluorescence intensity of RLS‐2 internalization was roughly one‐tenth of its binding, this implies that the amount of aptamer internalized by injured podocytes is likely much lower than their binding capacity. Interestingly, we observed that healthy podocytes could also uptake RLS‐2 at about one‐third of the level seen in injured podocytes. This may be attributed to shared molecules between injured and healthy podocytes that facilitate RLS‐2 internalization in healthy cells.

Aptamer‐drug conjugates (ApDCs), similar to antibody‐drug conjugates (ADC), are effective for targeted drug delivery.^[^ ^39^ ^]^ Generally, nanoparticles internalized through clathrin‐mediated endocytosis (CME) are believed to be transported to lysosomes,^[^ ^45^ ^]^ while cargo entering cells via caveolae‐dependent pathway (CvME) might bypass lysosomes^[^ ^46^ ^]^ and accumulate in the endoplasmic reticulum.^[^ ^47^ ^]^ Given that the endocytosis pathway of an aptamer‐drug conjugate is likely similar to that of the aptamer itself,^[^ ^48^ ^]^ we investigated RLS‐2′s entry into podocytes. Importantly, our study demonstrated that RLS‐2 efficiently internalizes into injured podocytes via caveolae‐mediated endocytosis and macropinocytosis. RLS‐2 was transported to lysosomes post‐endocytosis.^[^ ^49^ ^]^ Confocal microscopy confirmed that after internalization, RLS‐2 predominantly localizes to lysosomes. These findings suggest that RLS‐2 can be effectively used in ApDCs), particularly those requiring lysosomal processing for drug activation.^[^ ^50^ ^]^

Our in vivo biodistribution studies further support the renal specificity of RLS‐2. Interestingly, we observed a decrease in the kidney‐to‐liver ratio in RLS‐2 injection groups compared to the control aptamer. This finding was unexpected, as it suggests an increase in off‐target accumulation of RLS‐2 in the liver. However, lower kidney‐to‐liver ratios are a common issue in targeting glomerular delivery. In fact, the ratios reported in many other studies focusing on glomerular delivery are often less than 0.8, which is substantially lower than those observed in studies targeting the tubules.^[^ ^51^ ^]^ In our research, we observed a kidney‐to‐liver ratio of 1.13 ± 0.37 in mice with adriamycin‐induced nephropathy and a ratio of 2.72 ± 1.02 in mice with diabetic nephropathy. Despite the observed decrease, the kidney‐to‐liver ratio for RLS‐2 remains within a relatively high range for glomerular delivery. Additionally, no significant pattern differences were observed between the distribution of RLS‐2 and the control aptamer in liver and spleen tissues, suggesting that its distribution in these organs may be non‐specific.

However, we found that relying solely on the kidney‐to‐liver ratio was insufficient for assessing the effectiveness of podocyte‐targeted delivery. For instance, the kidney‐targeting peptide, which accumulates significantly in the kidney in vivo, predominantly distributes outside of glomeruli, rendering it unsuitable for podocyte targeting.^[^ ^36^ ^]^ Therefore, we utilized the mean fluorescence intensity ratio of glomerulus to tubule to evaluate the specific distribution of aptamers within glomeruli. Compared to the control aptamer, our study demonstrated a significant presence of RLS‐2 in glomeruli, particularly in the nephropathy group, where the ratio of glomerulus to tubule was two to three times higher than observed with the control aptamer. Additionally, we found that after tail vein injection of fluorophore‐conjugated RLS‐2, it co‐localized with the podocyte marker protein synaptopodin. Furthermore, in kidney cell suspensions from nephropathy mice, a higher fluorescence intensity ratio of podocyte to non‐podocyte was observed. These results further confirm the specific distribution of RLS‐2 in podocytes in vivo. These findings are noteworthy, as many studies focusing on podocyte delivery have not thoroughly characterized the specific distribution within glomeruli. Moreover, other studies involving small‐sized nucleic acid‐based nanomaterials have generally reported lower accumulation in glomeruli compared to tubules.^[^ ^52^, ^53^ ^]^

One legitimate concern is whether the observed accumulation of RLS‐2 in the tubular area indicates specific binding to tubules in vivo. Indeed, targeting materials intended for podocytes in vivo are designed small enough (typically less than 6 nm) to pass through the glomerular filtration membrane.^[^ ^54^ ^]^ Once filtered, such ultra‐small vectors can easily accumulate in the proximal tubules due to their active endocytic activity.^[^ ^55^ ^]^ As a short strand of DNA, the size of an aptamer usually measures less than 2 nm,^[^ ^56^ ^]^ making it inevitable for RLS‐2 to be reabsorbed. Consistently, we observed the accumulation of both RLS‐2 and scrambled RLS‐2 within tubular cells, rather than in the filtrate. Nevertheless, we believe that this will not compromise the targeting of RLS‐2 to glomeruli.

In our research, EPB41L5 was identified as a target protein of RLS‐2. EPB41L5 is a focal adhesion component highly expressed in podocytes, where it directly regulates actomyosin contractility, influencing focal adhesion maturation, cell spreading, and migration.^[^ ^57^ ^]^ In an adriamycin nephropathy mouse model, EPB41L5 was found to be significantly upregulated in a compensatory manner,^[^ ^58^ ^]^ which may explain the stronger binding ability of RLS‐2 to injured podocytes. Aptamer pull‐down assays demonstrated enrichment of EPB41L5 in the RLS‐2 pull‐down sample, and confocal laser microscopy revealed its co‐localization with RLS‐2. However, it is worth noting that the Coomassie Brilliant Blue staining revealed multiple protein bands, leading us to suspect that EPB41L5 may not be the only binding protein.

We successfully achieved partial silencing of *Epb41l5* and *Mmp10* with a 24 h AsiC treatment, resulting in mRNA levels being reduced by ≈40% and 30%, respectively. These two genes were selected as target for knockdown because the former encodes the RLS‐2 target protein, while knockdown of the latter can delay the progression of proteinuria.^[^ ^59^ ^]^ These results demonstrate that the AsiCs based on RLS‐2 successfully delivered siRNA into cells and downregulated gene expression without the need for transfection reagents. Although the silencing efficiency was relatively low, this result aligns with the study by Jeong et al., which reported that siRNA delivery mediated by Apt‐siRNA alone typically did not induce significant gene silencing in cells.^[^ ^60^ ^]^ They suggested that co‐delivery of an endosomal escape agent was necessary to enhance siRNA endosomal release and cytoplasmic delivery. Therefore, further optimization of RLS‐2 targeting strategies could enhance its therapeutic potential.

In frozen kidney sections, we observed strong nuclear binding of RLS‐2, whereas this phenomenon was not observed in live cells. Although the mechanism underlying nuclear binding remains unclear, this finding suggests that when RLS‐2 directly interacts with tissue section samples, it is more likely to bind strongly and nonspecifically to the nuclei of all cells.

Given the complexities of drug delivery to podocytes, the development of RLS‐2 as a podocyte‐specific targeting agent represents a significant advancement. While further optimization is required, RLS‐2 provides a foundation for the development of more effective, safer therapeutic strategies for podocyte‐related diseases.

## 4 Experimental Section

### Cell Culture

The immortalized mouse podocyte cell line (MPC5) was donated by Professor Peter Mundel from Goldfinch Bio. Human mesangial cells (HMC) and human proximal tubular epithelial cells (HK‐2) were purchased from the Chinese Academy of Sciences (Shanghai, China). Human renal glomerular endothelial cells (HRGEC) were purchased from ScienCell Research Laboratories (Carlsbad, USA). Human podocyte cells (HPC) were purchased from OTWO Biotech (Guangzhou, China).

The MPC5 and HPC cell lines were cultured at 33 °C in RPMI 1640 supplemented with 10% fetal bovine serum (FBS, Gibco, USA) and 10 U mL^−1^ mouse recombinant interferon‐γ (#485‐MI, R&D Systems). To achieve full differentiation, the podocytes were cultured at 37 °C in RPMI 1640 (Corning, USA) with 10% FBS for 10–14 days prior to their use in modeling or other experiments. Adriamycin (#S1208, Selleck Chemicals, 0.3 µg mL^−1^), puromycin aminonucleoside (#HY‐15695, MedChemExpress, 30 µg mL^−1^), or glucose (#G7021, Sigma–Aldrich, 40 mmol L^−1^) were added to the medium for 48 h to induce different podocyte injury models. HRGECs were cultured at 37 °C in DMEM/F12 (Corning, USA) with 10% FBS. HK‐2 cells were cultured at 37 °C in RPMI 1640 with 10% FBS, and human mesangial cells were cultured at 37 °C in DMEM (Corning, USA) supplemented with 10% FBS.

### ssDNA Library and Primers for Cell‐SELEX

The initial ssDNA library was synthesized by Sangon Biotechnology Co., Ltd (Shanghai, China) with the following sequence: 5′‐GTT CGT GGT GTG CTG GAT GT ‐N (36)‐ TGA CAC ATC CAG CAG CAC GA‐ 3′, where N represents a randomized nucleotide. For PCR amplification and subsequent single‐strand DNA separation, a fluorescence‐labeled forward primer (5′‐FAM‐GTT CGT GGT GTG CTG GAT GT‐3′) and a reverse primer containing a poly A tail and spacer 18 (5′‐AAA AAA AAA AAA AAA AAA A /iSp18/ TCG TGC TGC TGG ATG TGT CA‐3′) were also synthesized.

### Cell‐SELEX

The initial library or 100 pmol of aptamers from the final round of selection were first denatured at 95 °C for 10 min, followed by cooling on ice for 10 min. After washing the cells with a washing buffer containing 2 g L^−1^ glucose and 5 mm MgCl_2_ in DPBS, the aptamers were diluted to 1 mL with binding buffer containing 1 mg mL^−1^ bovine serum albumin (BSA), 1 mg mL^−1^ yeast tRNA (#R8759, Sigma–Aldrich), and 0.1 mg mL^−1^ salmon sperm DNA (#D7656, Sigma–Aldrich) in the washing buffer. This solution was then incubated with normal podocytes on ice for 1 h, followed by incubation with injured podocytes for another hour. After incubation, the bound aptamers were collected and PCR‐amplified (15 cycles of 30 s at 98 °C, 20 s at 60 °C, and 20 s at 72 °C, followed by a final extension of 3 min at 72 °C). The PCR product was concentrated to 100 µL using n‐butanol, mixed with 2×TBE DNA loading buffer, and subjected to denaturing PAGE. Under blue light, the gel containing FAM‐labeled target ssDNA showing green fluorescence was excised into small pieces. These pieces were then heated with water for 20 min, the gel was removed by filtration, and the supernatant concentrated and dialyzed. The dialysis product containing separated single‐strand DNA was used for the subsequent round of selection.

To increase the stringency of the selection, the number of normal podocytes used in the process was increased from 1 × 10^6^ to 5 × 10^6^, while the number of injured podocytes was decreased from 5 × 10^6^ to 1 × 10^6^. The incubation time with normal podocytes was extended from 1 to 2 h, whereas the incubation time with injured podocytes was reduced from 1 h to 30 min. Additionally, the washing duration was extended from 1 to 5 min. High‐throughput sequencing was performed to identify the sequences of the selection products.

### Secondary Structure Prediction

The aptamer secondary structure was predicted using mfold web server.^[^ ^61^ ^]^ The parameters, including temperature, sodium, and magnesium concentration were altered to the situation of Cell‐SELEX.

### Flow Cytometry Analysis

In the monitoring of the screening process, podocytes or human‐derived kidney cell lines were treated with 0.02% EDTA for digestion. Subsequently, 3 × 10^5^ cells were incubated with FAM‐labeled aptamer (concentration of 250 nM in binding buffer) at 4 °C for 1 h. Post‐incubation, the cells underwent two washes before being subjected to flow cytometry analysis using a Beckman instrument (USA). FlowJo software was employed for data analysis.

The dissociation constant (K_d_) of the aptamers was determined as follows: Podocytes were incubated with varying concentrations (0, 10, 25, 50, 100, 200, 400, and 600 nM) of the library or the specific aptamer under study. Flow cytometry was then used to measure fluorescence intensity. GraphPad Prism 9 software was used for curve fitting, employing the equation: Y = B_max_X/(K_d_ + X), where “X” denotes aptamer concentration, “Y” the specific mean fluorescence intensity, and “B_max_” the maximum specific fluorescence intensity. The specific mean fluorescence intensity was calculated by subtracting the control aptamer's average fluorescence intensity from that of the test sample.

In the competitive binding assay, cells treated with 0.02% EDTA were incubated with various formulations: 250 nM FAM‐library, 250 nM FAM‐S7, 250 nM FAM‐RLS‐2, 2.5 µM Library + 250 nM RLS‐2, and 2.5 µM RLS‐2 + 250 nM FAM‐S7. Subsequent to incubation, cells were washed and analyzed using flow cytometry as described above.

To assess the impact of temperature on aptamer binding, podocytes were exposed to 250 nM aptamers at either 4 or 37 °C prior to flow cytometry analysis.

For investigating cellular uptake of aptamers, cells were switched to a corresponding serum‐free culture medium before incubation. Time‐dependent cellular uptake was examined by adding cooled, denatured FAM‐labeled RLS‐2 and scrambled RLS‐2 to a final concentration of 250 nM. Cells were incubated at 37 °C for varying durations (5, 14, 30, 60, 90, 120, and 150 min), followed by treatment with 0.25% trypsin and 0.02% EDTA to thoroughly remove surface binding. Flow cytometry was then conducted for analysis. A similar approach was taken for assessing concentration‐dependent uptake, using incubation times of 1 h with FAM‐labeled RLS‐2 and scrambled RLS‐2 at concentrations ranging from 25 to 1000 nM.

To elucidate the internalization pathway of the aptamers, podocytes were pre‐treated with amiloride (#HY‐B0285A, MedChemExpress), genistein (#HY‐14596, MedChemExpress), and chlorpromazine (#HY‐B0407A, MedChemExpress) at concentrations of 40, 70, and 100 nM for 30 min at 37 °C before aptamer incubation. A control group was treated with 0.1% DMSO. Additionally, to determine if aptamer entry into cells was energy‐dependent, a group of podocytes was incubated with aptamers at 4 °C.

For binding ability assessment with freshly isolated kidney cells, the upper and lower poles of the kidney were dissected with scissors and then digested with a solution of collagenase XI (#C7657, Sigma–Aldrich, 0.15 mg mL^−1^), hyaluronidase (#HY‐107910, MedChemExpress, 30 µg mL^−1^) and collagenase I‐S (#C1639, Sigma–Aldrich, 2 mg mL^−1^) at 37 °C. The cell suspension was then filtered sequentially through 70 and 40 µm cell strainers and incubated for 5 min at room temperature in red blood cell (RBC) lysis buffer (NH_4_Cl, KHCO_3_, ddH_2_O). After centrifugation, cells were resuspended and incubated with 1µM FAM labeled aptamer and 4 µg mL^−1^ PE‐labeled podocalyxin antibody (#12‐8883‐82, Thermo Fisher Scientific), with the presence of 1 mg mL^−1^ salmon sperm DNA and 1% BSA at room temperature for 30 min. After washing, cells were then stained with fixable dye (#423 105, Biolegend, 1:1000 dilution in PBS) at room temperature for 15 min. Cells were then washed with 5% BSA PBS solution and resuspended in PBS for flow cytometry. The gating stratagem is shown in Figure S13 (Supporting Information).

### Confocal Imaging

For the binding assay, podocytes were cultured in dishes with glass bottoms. The cells were incubated with fluorescently labeled aptamer (250 nM in binding buffer) at 4 °C for 1 h. Subsequently, they were treated with Hoechst 33 342 dye (5 µg mL^−1^ in washing buffer) for nuclear staining. To visualize the cellular transport, cells were incubated with aptamers at 37 °C, followed by incubation with LysoTracker (#L7526, Thermo Fisher Scientific) and ER Tracker (#C1042S, Beyotime) for 30 min prior to imaging. Post‐washing, imaging was performed using a Nikon A1 Ti laser scanning confocal microscope.

In immunofluorescence staining, 4‐µm thick frozen kidney sections or cells on coverslips were fixed with 4% paraformaldehyde and blocked using 5% bovine serum albumin for 1 h at room temperature. Then the samples were permeabilized with 0.3% Triton X‐100 for 5 min and then incubated overnight at 4 °C with primary antibodies as shown in Table S7 (Supporting Information). Subsequently, the samples were washed and stained with appropriate secondary antibodies. Fluorescence images were captured using a Nikon A1 Ti laser scanning confocal microscope.

### Aptamer Stability Analysis

For stability analysis, aptamers were dissolved in water to achieve a final concentration of 100 µm. Then, 50 µL of this aptamer solution was mixed with 450 µL of cell culture medium containing 10% FBS. This mixture was incubated at 37 °C. At predetermined time points (2, 4, 6, 8, 10, 12, 24, 36, and 48 h), 45 µL samples were collected and stored at −80 °C. Subsequently, all samples were subjected to agarose gel electrophoresis for analysis.

### Cytotoxicity Experiment

2 × 10^3^ MPC5 cells were plated in a 96‐well plate and cultured for three days. After cultivation, these cells were exposed to specific chemicals to induce injury. After 24 h, denatured and cooled aptamers were introduced into the medium at varying concentrations (0, 250, 500, and 1000 nM) to treat the cells for an additional 24 h period. Cell viability was assessed using the Cell Counting Kit‐8 (MedChemExpress, USA). Absorbance at a wavelength of 450 nm was measured using a microplate reader, and cell viability was calculated using the formula: (A_Sample_ ‐A_Blank_)/(A_0 nM_‐A_Blank_) ×100%.

### Animal Modeling

The animal experiment was approved by the Animal Ethics Committee of Zhejiang University. Mice were obtained from Slac Laboratory Animal Co., Ltd (Shanghai, China) and underwent one week of acclimatization before the experiments. Subsequently, they were randomly divided into nephropathy and control groups.

For the adriamycin‐induced nephropathy model, male BALB/c mice (8 weeks old) received a tail vein injection of 15 mg kg^−1^ adriamycin and were observed for an additional 2 weeks. The control group was administered an equivalent volume of 0.9% saline. In the STZ‐induced diabetic nephropathy model involving DBA/2 mice (8 weeks old), fasting was enforced for 6 h prior to the experiment. Streptozotocin (Selleck Chemicals, USA) was freshly prepared in sodium citrate buffer (0.05 M, pH 4.5) at a concentration of 7.5 mg mL^−1^. The nephropathy group received intraperitoneal injections of 40 mg kg^−1^ streptozotocin daily for 5 consecutive days, while the control group received sodium citrate buffer. After injection, all mice had access to 10% sucrose solution to mitigate hypoglycemia risks. Two weeks following the final injection, mice exhibiting venous blood glucose levels above 16 mm were retained for an additional 2 months. All mice were housed under controlled conditions with free access to food, at 25 °C temperature and 50% humidity.

### In Vivo Biodistribution

In this study, mice were administered Cy5‐labeled aptamers (0.16 nmol g^−1^ body weight) via tail vein injection. One‐hour post‐injection, the mice were euthanized, and major organs (heart, lungs, liver, spleen, kidneys) were harvested for weight measurement and fluorescence visualization. Imaging was performed using the Lumina LT Series III system (PerkinElmer, USA), and image analysis was conducted with LivingImage software (PerkinElmer, USA).

### Blood and Urine Chemistry

Blood glucose levels were quantified using a GA‐3 glucose meter (Sinocare, China). Urine albumin concentrations were determined employing assay kits from Chondrex (#3012, USA), and urine creatinine concentrations were measured using kits from Cayman (#500 701, USA).

### Pathology and Immunofluorescence Assay

For histopathological analysis, kidney tissues were embedded in paraffin, and sections of 4‐µm thickness were prepared for periodic acid‐Schiff (PAS) and Masson's trichrome staining.

### Aptamer Pulldown Assay

High‐glucose‐induced damaged podocytes were dissociated from the culture dish using EDTA, and ≈ 450 µL of wet podocyte pellet was obtained. Following the manufacturer's instructions, the total membrane protein fraction was extracted using the Plasma Membrane/Protein Isolation and Cell Fractionation Kit (#SM‐005, Invent) and solubilized in 800 µL of lysis buffer (DPBS + 5 mM MgCl₂ + 1% Triton X‐100). To inhibit protein degradation, 10 mm PMSF and Protease Inhibitor Cocktail were added just before use. After extraction, 50 µL of the protein sample was reserved as input, and the remaining protein was divided into three equal portions. Each portion was incubated with 1 mL of Binding Buffer containing 1 mg mL^−1^ salmon sperm DNA at 4 °C for 1 h. Then, 750 µL of a solution, either without the aptamer or containing 2 µm aptamer, was added to each group, and the mixtures were incubated at 4 °C for 1 h to capture the binding proteins. Each group was further incubated with 70 µL of Streptavidin Agarose 6FF (#HY‐K0218A, MedChemExpress) that had been pre‐blocked with 5% BSA at 4 °C for 2 h. After thorough washing, the supernatant was discarded, and the beads were heated with Laemmli Sample Buffer (working concentration, #1 610 747, Bio‐Rad) at 95 °C for 5 min to obtain protein samples. All protein samples were separated by SDS‐PAGE, followed by Coomassie Brilliant Blue staining for protein visualization. The corresponding gel lanes were excised for LC‐MS/MS analysis.

### Chimera Synthesis

The RLS‐2‐sense and antisense strands were synthesized as shown in Table S6 (Supporting Information). The powders were dissolved in DEPC‐treated water to a concentration of 20 µm. After mixing in equal volumes, 5 × annealing buffer (50 mm HEPES, 750 mm NaCl, 5 mm CaCl₂, 5 mm MgCl₂, and 13.5 mm KCl) was added to achieve a final concentration of 1 ×. The mixture was then heated to 95 °C for 10 min in a PCR machine, followed by a gradual cooling to 25 °C over 1 h to form AsiCs.

### qRT‐PCR

RNA was extracted using an RNA extraction kit (#RN001, ESScience) and the concentration was determined using Nanodrop 2000 (Thermo Fisher Scientific, USA). 1 µg of total RNA was reverse‐transcribed into complementary DNA (cDNA) using the HiScript II Q RT SuperMix qPCR kit (#R201, Vazyme). The primer sequences for qPCR are listed in Table S8 (Supporting Information). RT‐qPCR was performed according to the kit instructions with ChamQ SYBR qPCR Master Mix (#Q341‐02, Vazyme) on a Bio‐Rad CFX‐96 real‐time PCR system, with each sample run in triplicate. Glyceraldehyde‐3‐phosphate dehydrogenase (*Gapdh*) was used as the normalizing gene. RT‐qPCR data were represented as fold differences by the 2^−ΔΔCt^ method.

### Statistical Analysis

GraphPad Prism was used to generate graphs and perform statistical analysis. The values presented represent the mean ± standard deviation (SD) from at least three independent experiments. For comparisons between two groups, a two‐tailed Student's t‐test was applied. For comparisons among multiple groups, a one‐way ANOVA followed by Tukey's multiple comparisons test was performed for pairwise comparisons. A *P* value of less than 0.05 was considered statistically significant.

## Conflict of Interest

The authors declare no conflicts of interest.

## Supporting information



Supporting Information

## Data Availability

The data that support the findings of this study are available from the corresponding author upon reasonable request.;
