# Supplementary material for: Screening and Identification of Novel DNA Aptamer for Targeted Delivery to Injured Podocytes in Glomerular Diseases
Source: Adv Sci (Weinh). 2025 Apr 3;12(20):2412356. doi: 10.1002/advs.202412356 (PMC12120727; doi:10.1002/advs.202412356)
Supplement: Supplementary file 1 — Supporting Information [file ADVS-12-2412356-s001.docx]

**SUPPLEMENTARY INFORMATION FOR**

**Title**

Screening and Identification of Novel DNA Aptamer for Targeted Delivery to Injured Podocytes in Glomerular Diseases

**Authors**

Chao Zhou, Zhaofeng Luo, Zheng Zhang, Qing Ye, Dongjie Wang, Hanyan Meng, Jiayu Zhang, Shifan Zhu, Lidan Hu, Jianhua Mao

**This PDF file includes**

Supplementary Tables 1-8

Supplementary Figures 1-13 and Legends

**Supplementary Tables**

**Table S1. The Sequences of Candidate Aptamers.**

| **Name** | **Sequence composition** |
| --- | --- |
| S1 | *GTTCGTGGTGTGCTGGATGT*ACCCAATAACGAGTCGGGCATCCCTGTTGGGGCTAT*TGACACATCCAGCAGCACGA* |
| S2 | *GTTCGTGGTGTGCTGGATGT*TGCACCGTCAACCCTCGATATATCGCTGTGTCTTTT*TGACACATCCAGCAGCACGA* |
| S3 | *GTTCGTGGTGTGCTGGATGT*CGCAACAATTGCCACTTAAATTGTTCTAAACGTCGC*TGACACATCCAGCAGCACGA* |
| S4 | *GTTCGTGGTGTGCTGGATGT*CGACGGACTTATTCGTTGAATAATGCTAAATGTTGC*TGACACATCCAGCAGCACGA* |
| S5 | *GTTCGTGGTGTGCTGGATGT*TGGCGTGGGGCACAAAGCTGACGGACCTCTTGATGA*TGACACATCCAGCAGCACGA* |
| S6 | *GTTCGTGGTGTGCTGGATGT*CGGCAAACAATTCCGCGCGATTGTTACGGTATCCGG*TGACACATCCAGCAGCACGA* |
| S7 | *GTTCGTGGTGTGCTGGATGT*CGGTGCGGTTAGGTTTGGTGCGGGGGGGTAATTCGA*TGACACATCCAGCAGCACGA* |
| The primer region sequences are indicated in italics. | |

**Table S2. The Sequences of S7 Variants and RLS-1 to RLS-4.**

| **Name** | **Sequence composition** |
| --- | --- |
| S7 | *GTTCGTGGTGTGCTGGATGT*CGGTGCGGTTAGGTTTGGTGCGGGGGGGTAATTCGATGAC*ACATCCAGCAGCACGA* |
| S7-1 | *GTTCGTGGTGTGCTGGATGT*CGGTGCGGTTAGGTTTGGTGCGGGGGGGTTTCTCGATGAC*ACATCCAGCAGCACGA* |
| S7-2 | *GTTCGTGGTGTGCTGGATGT*CGGTGCGATTAGGTTTGGTGCGGGGGGGTAATTCGATGAC*ACATCCAGCAGCACGA* |
| S7-3 | *GTTCGTGGTGTGCTGGATGT*CGGTGCGGTTAGGTTCGGTGCGGGGGGGTAATTCGATGAC*ACATCCAGCAGCACGA* |
| RLS-1 | GTCGTTAGGTTTGGTGCGGGGGGGTAACGAC |
| RLS-2 | TCGTTAGGTTTGGTGCGGGGGGGTAACGA |
| RLS-3 | CGTTAGGTTTGGTGCGGGGGGGTAACG |
| RLS-4 | GTTAGGTTTGGTGCGGGGGGGTAAC |
| Scrambled RLS-2 | GTAGGTATGGAAGGTGGTTCGCTGTGGCG |
| The primer region sequences are indicated in italics. The nucleotides replaced based on S7 were shown in red. | |

**Table S3. The Sequences of Aptamers with Different Modifications.**

| **Name** | **Sequence composition** |
| --- | --- |
| PS-RLS-2 | T*C*G*TTAGGTTTGGTGCGGGGGGGTAA*C*G*A |
| 2’-F-RLS-2 | *UC*G*UU*AGG*UUU*GG*U*G*C*GGGGGGG*U*AA*C*GA |
| 2’-O-Me-RLS-2 | *TCG*TTAGGTTTGGTGCGGGGGGGTAA*CGA* |
| Bio-Scrambled RLS-2 | biotin-T(6)-GTAGGTATGGAAGGTGGTTCGCTGTGGCG |
| Bio-RLS-2 | biotin-T(6)-TCGTTAGGTTTGGTGCGGGGGGGTAACGA |

The asterisk (*) represents phosphorothioate modifications in the backbone, the underlined nucleotides indicate 2'-fluoro modifications, and the italicized nucleotides represent 2'-O-methyl modifications.

**Table S4. Group Characteristics of the Experimental Animals.**

|  | **Adriamycin nephropathy** | |
| --- | --- | --- |
|  | **Control (Solvent injection)** | **Adriamycin injection** |
| **Strain** | BALB/c | |
| **Body weight (g)** | 25.37 ± 1.18 | 20.85 ± 1.07^***^ |
| **Blood glucose (mM)** | - | - |
| **Kidney weight (g)** | 0.43 ± 0.02 | 0.39 ± 0.03^*^ |
| **KW/BW (g/100 g)** | 1.71 ± 0.08 | 1.86 ± 0.11^*^ |

|  | **STZ-induced diabetic nephropathy** | |
| --- | --- | --- |
|  | **Control (Solvent injection)** | **STZ** |
| **Strain** | DBA/2 | |
| **Body weight (g)** | 27.53 ± 0.86 | 17.13 ± 1.71^***^ |
| **Blood glucose (mM)** | 6.22 ± 0.87 | 26.07 ± 1.80^***^ |
| **Kidney weight (g)** | 0.52 ± 0.03 | 0.44 ± 0.03^***^ |
| **KW/BW (g/100 g)** | 1.89 ± 0.11 | 2.57 ± 0.13^***^ |

Mean ± SEM; *n* = 6; **P* < 0.05, ***P* <0.01, ****P*<0.001 versus control; STZ, streptozotocin; KW/BW, kidney weight/body weight.

**Table S5. Information of candidate proteins.**

| **Uniprot ID** | **Protein name (*Gene Abbreviation*)** | **Score (RLS-2)** | **Score (RLS-2) / Score (Scrambled-RLS-2)** |
| --- | --- | --- | --- |
| Q8BMK4 | Cytoskeleton-associated protein 4 (*Ckap4*) | 56.68 | 3.88 |
| F8VPU2 | FERM, ARHGEF and pleckstrin domain-containing protein 1 (*Farp1*) | 36.55 | 3.47 |
| Q9JKF1 | Ras GTPase-activating-like protein IQGAP1 (*Iqgap1*) | 35.24 | 3.23 |
| Q811D0 | Disks large homolog 1 (*Dlg1*) | 34.99 | 3.28 |
| Q8BGS1 | Band 4.1-like protein 5 (*Epb41l5*) | 33.56 | 16.78 |
| Q99P72 | Reticulon-4 (*Rtn4*) | 30.61 | 12.70 |
| O70318 | Band 4.1-like protein 2 (*Epb41l2*) | 30.45 | 12.43 |
| Q91ZX7 | Sodium/potassium-transporting ATPase subunit beta-1 (*Lrp1*) | 27.35 | 13.68 |
| P13808 | Anion exchange protein 2 (*Slc4a2*) | 26.87 | 13.44 |
| O35245 | Polycystin-2 (*Pkd2*) | 26.66 | 13.33 |
| Q9QXN0 | Protein Shroom3 (*Shroom3*) | 26.49 | 13.25 |
| Q8VBZ3 | Putative lipid scramblase CLPTM1 (*Clptm1*) | 24.52 | 4.24 |
| Q8BVD5 | MAGUK p55 subfamily member 7 (*Mpp7*) | 21.48 | 4.81 |
| P14094 | Sodium/potassium-transporting ATPase subunit beta-1 (*Atp1b1*) | 20.02 | 9.02 |
| P84091 | AP-2 complex subunit mu (*Ap2m1*) | 17.18 | 8.59 |
| O54724 | Caveolae-associated protein 1 (*Cavin1*) | 17.15 | 6.19 |
| Q6P5F7 | Protein tweety homolog 3 (*Ttyh3*) | 16.31 | 8.16 |

**Table S6. Sequence Composition of AsiCs.**

| **Name** | **Sequence composition** |
| --- | --- |
| RLS-2-si*Epb415*-sense | TCGTTAGGTTTGGTGCGGGGGGGTAACGA**UUAACUACACUGACUUUGUACAU**TT |
| Scrambled RLS-2-si*Epb41l5*-sense | GTAGGTATGGAAGGTGGTTCGCTGTGGCG**UUAACUACACUGACUUUGUACAU**TT |
| si*Epb415*-antisense | **AUGUACAAAGUCAGUGUAGUU**TT |
| RLS-2-si*Mmp10*-sense | TCGTTAGGTTTGGTGCGGGGGGGTAACGA**UUCCAGCUAACUUCCACCUUU**TT |
| si*Mmp10*-antisense | **AAAGGUGGAAGUUAGCUGG**TT |

The ribonucleotides are highlighted in bold.

**Table S7. Antibody Used in This Research.**

| **Name** | **Producer** | **Catalogue number** | **Dilution** |
| --- | --- | --- | --- |
| Anti-NPHS2 antibody | Abcam | ab50339 | 1:1000 |
| Synaptopodin Antibody (D-9) | Santa Cruz Biotechnology | sc-515842 | 1:100 |
| EPB41L5 Antibody (F-9) | Santa Cruz Biotechnology | sc-515440 | 1:100 |
| AQP1 Polyclonal antibodies | Proteintech | 20333-1-AP | 1:1000 |
| TRA-1-81 (Podocalyxin) Monoclonal Antibody (TRA-1-81), PE | Thermo Fisher Scientific | 12-8883-82 | 1:50 |

**Table S8. Sequence Composition of the Primers Used for qPCR.**

| **Primer** | **Sequence composition** |
| --- | --- |
| Gapdh-F | GACTTCAACAGCAACTCCCAC |
| Gapdh-R | TCCACCACCCTGTTGCTGTA |
| Epb41l5-F | ATCTTCGTGAGGAGCTAACCC |
| Epb41l5-R | GCCAAGTTCAGCTTGCAGATTA |
| Mmp10-F | TTGCAGTTGGAGAACACGGA |
| Mmp10-R | TAGCTGGGCTTGTGGAGAAC |

**Supplementary Figures**

**Figure S1**


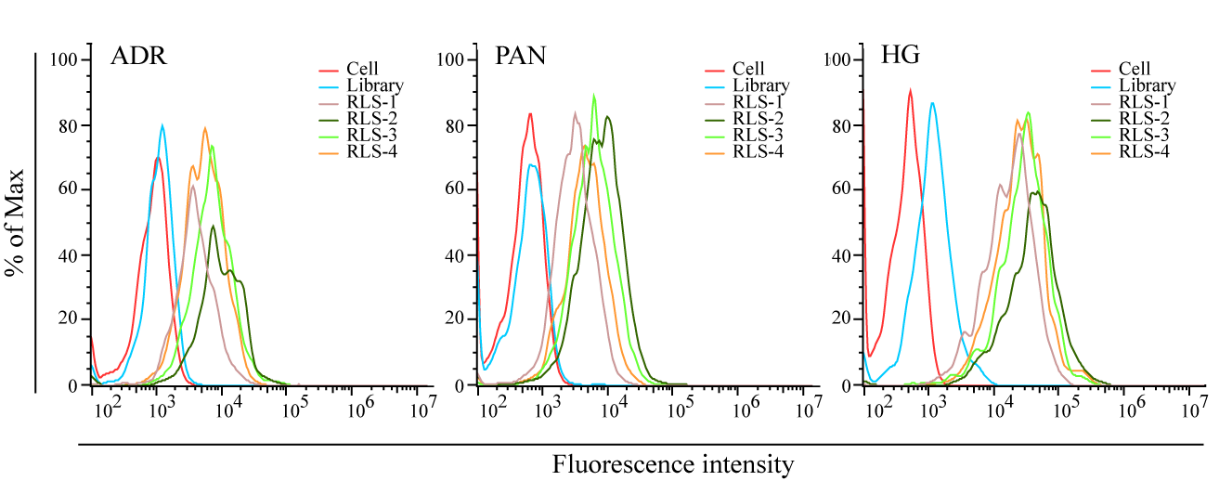


**Binding of Different S7-2 Truncation Forms to Injured Podocytes Analyzed by Flow Cytometry.** Injured MPC5 cells were detached using 0.02% EDTA and incubated with RLS-1~4 at 37°C for 1 hour. FAM-labeled library or untreated cells were used as controls.

**Figure S2**

**
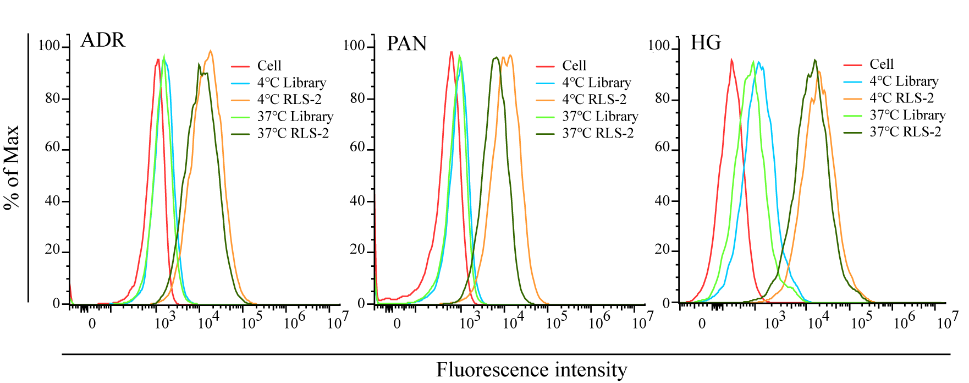
**

**The Binding of RLS-2 to Injured Podocytes at Different Temperature Analyzed by Flow Cytometry.** Injured MPC5 cells were detached using 0.02% EDTA and incubated with 250 nM RLS-2 at 4°C or 37°C for 1 hour. FAM-labeled library or untreated cells were used as control.

**Figure S3**


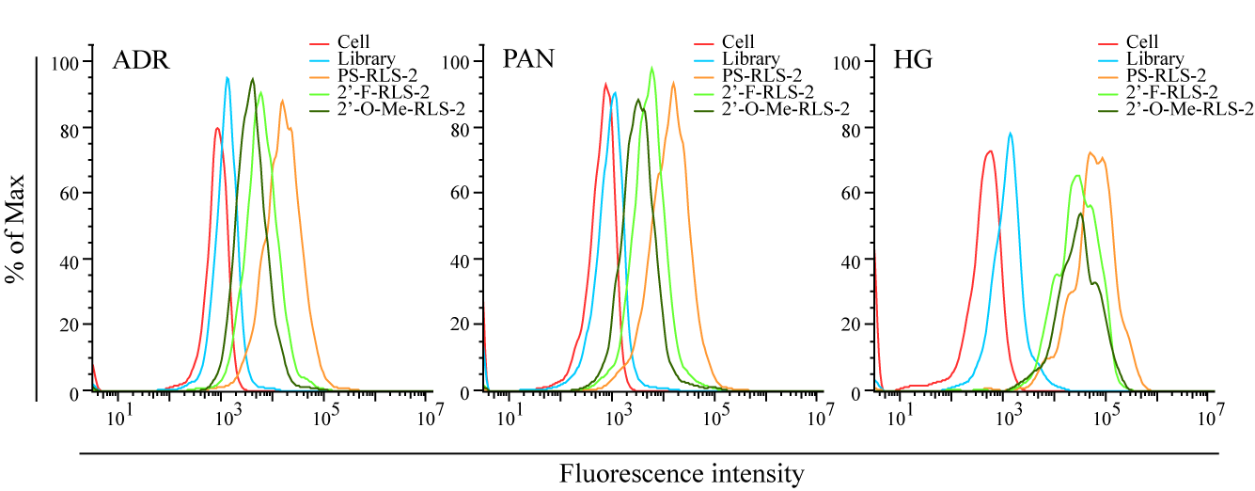


**Binding of Modified RLS-2 to Injured Podocytes Analyzed by Flow Cytometry.** Injured MPC5 cells were detached using 0.02% EDTA and incubated with phosphorothioate-, 2'-fluoro-, and 2'-O-methyl-modified RLS-2 at 37°C for 1 hour. FAM-labeled library or untreated cells were used as controls.

**Figure S4**

**
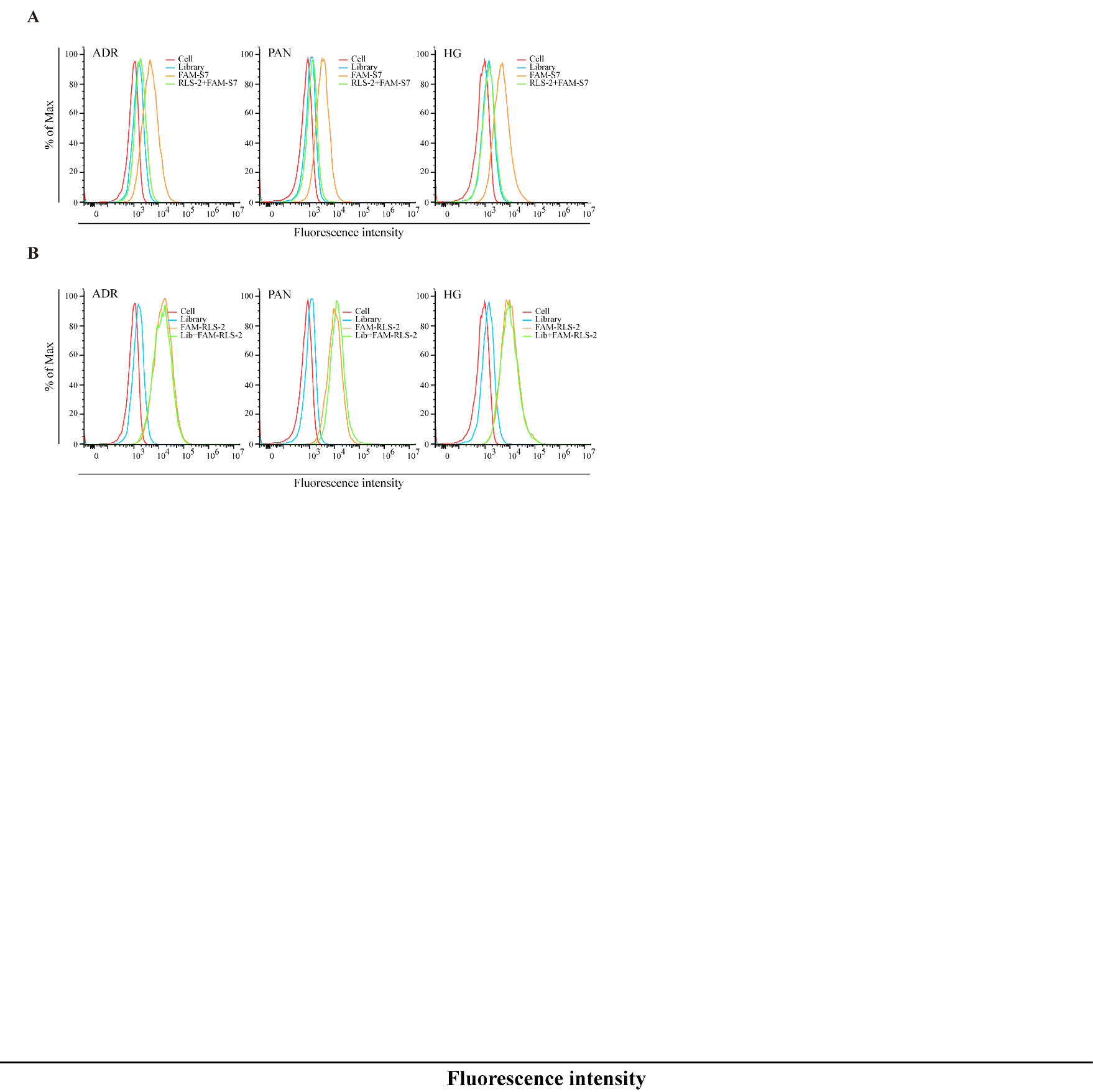
**

**RLS-2 Competes with S7 for Binding to Injured Podocytes. (A)** Flow cytometry results of injured podocytes after incubation with 250 nM FAM-S7 or a mixture of 250 nM FAM-S7 and 2.5 μM RLS-2 at 4°C. **(B)** Flow cytometry results of injured podocytes after incubation with 250 nM FAM-RLS-2 or a mixture of 250 nM FAM-RLS-2 and 2.5 μM library at 4°C. FAM-labeled library or untreated cells were used as controls**.**

**Figure S5**

**
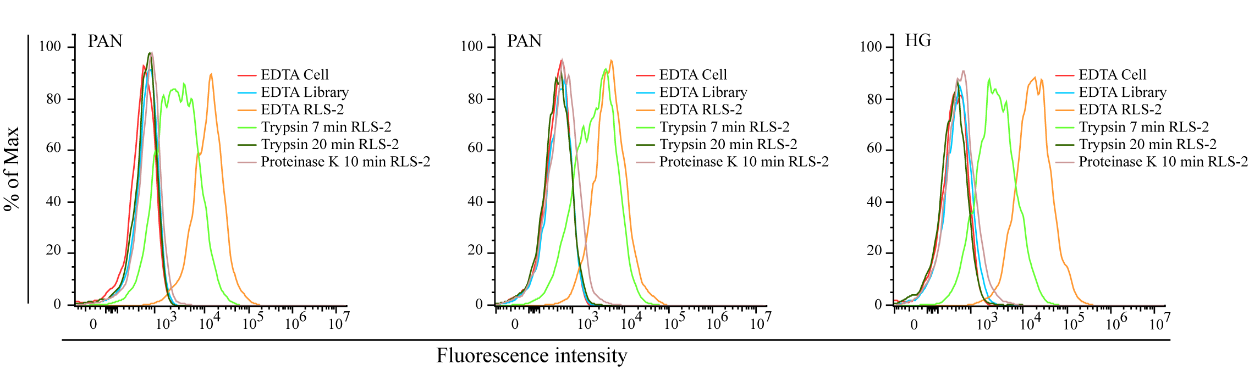
**

**RLS-2 Binds to Protein Targets on the Podocyte Surface.** Injured MPC5 cells were pretreated with 0.25% trypsin or 1% proteinase K at 37°C before being incubated with 250 nM RLS-2 at 4°C for 1 hour. Treatment with 0.02% EDTA was used as a control.

**Figure S6**

**
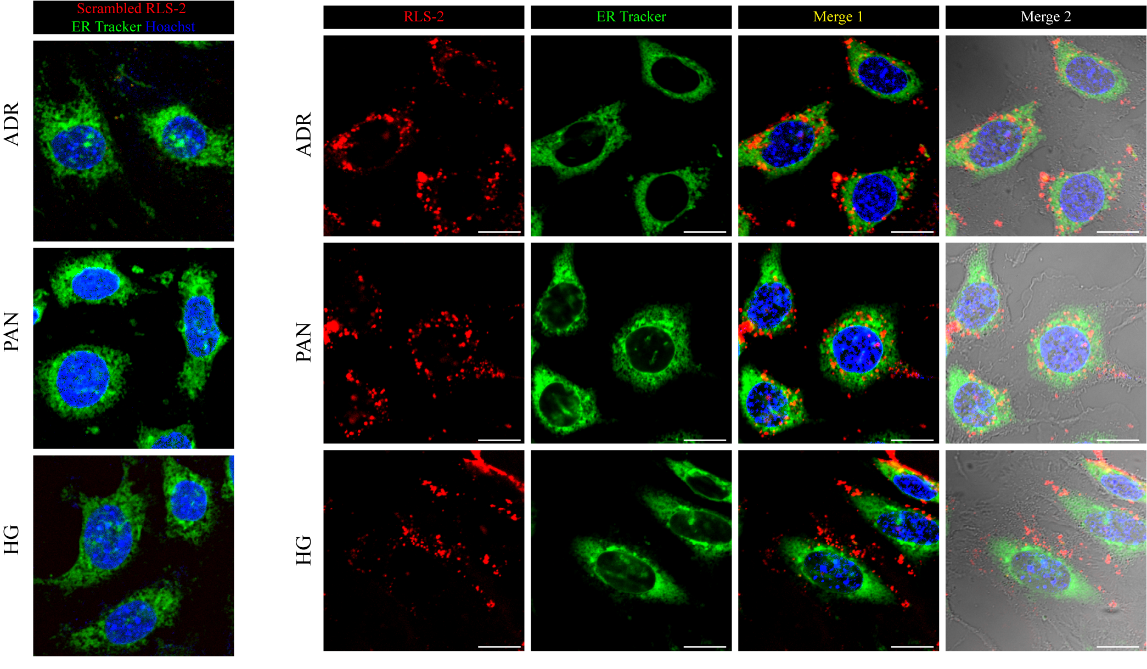
**

**RLS-2 Does Not Co-Localize with the Endoplasmic Reticulum in Cells.** Representative confocal images showing the distribution of RLS-2 (Cy5 labeled, red) and its co-localization with an endoplasmic reticulum marker (ER-Tracker, green) in injured MPC5 cells after incubation for 60 min at 37°C. Nuclei were counterstained with Hoechst 33342 (blue). Scale bars, 10 μm.**Figure S7**

**
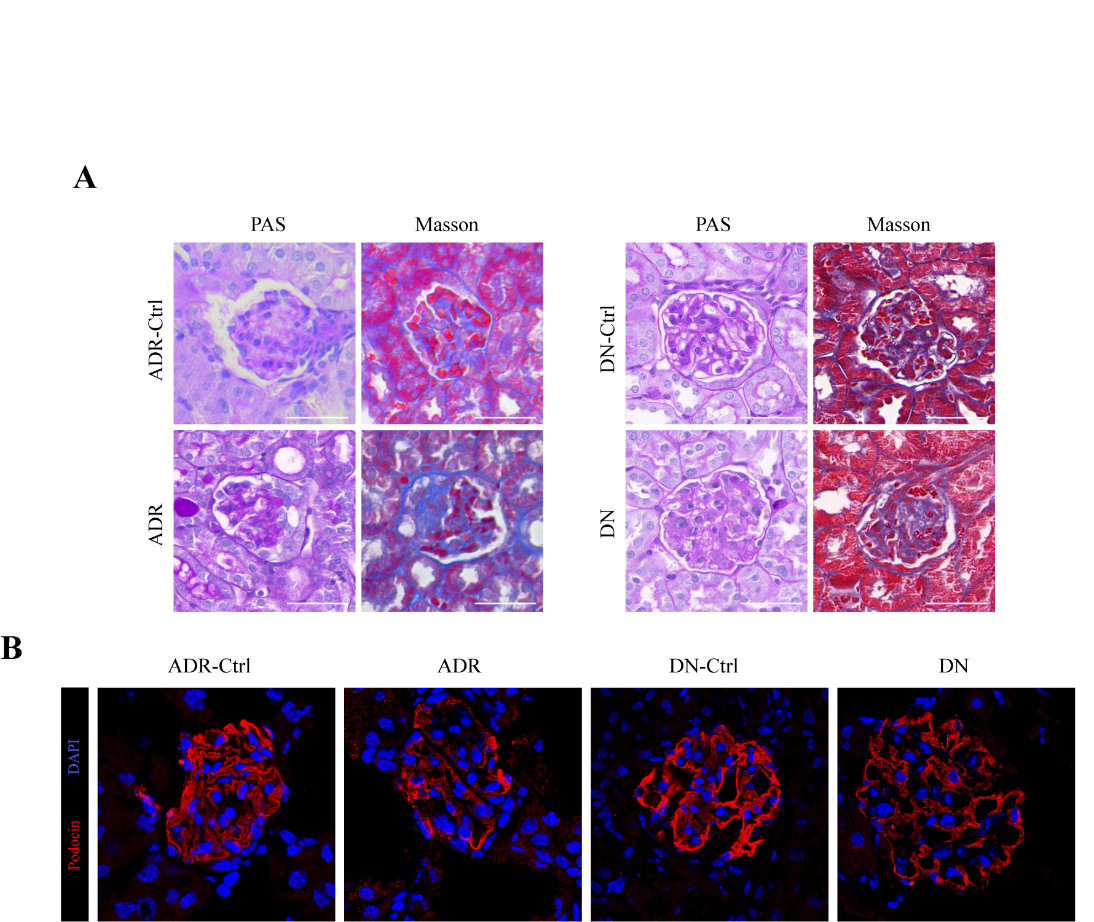
**

**Renal Histomorphological Examination Confirmed Glomerular Lesions in Nephropathy Models.** (A) Kidney sections stained with Periodic acid–Schiff (PAS) and Masson’s trichrome staining. Scale bars, 50 μm. (B) Representative confocal microscopy images showing podocin staining (red) in glomeruli of different mouse groups. Nuclei were counterstained with DAPI (blue). Scale bars, 50 μm.

**Figure S8**


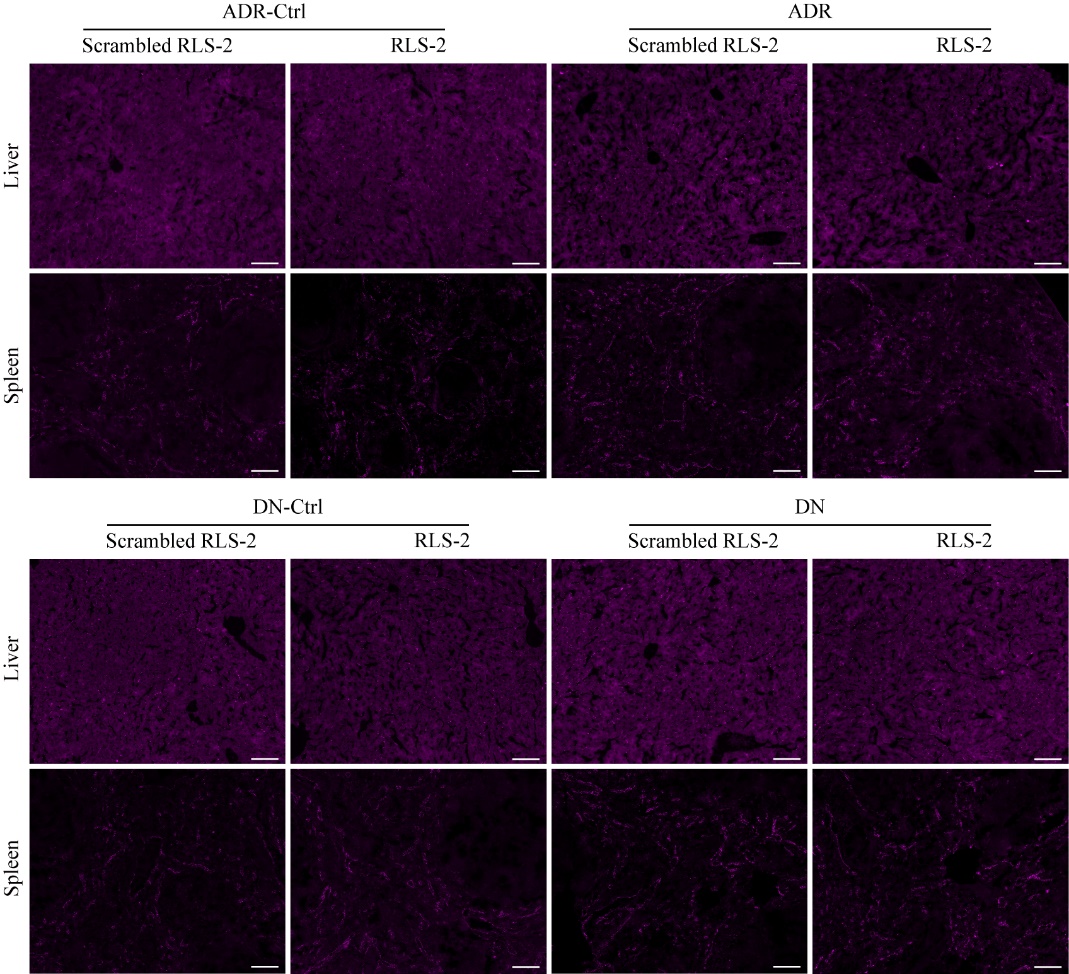


**Distribution of Aptamers in Mouse Liver and Spleen.** Mice were euthanized 1 hour after tail vein injection of Cy5-labeled aptamers at a dose of 0.16 nmol/g body weight. Frozen sections of the liver and spleen were prepared, and the distribution of the aptamers was observed using fluorescence microscopy. Scrambled RLS-2 was used as the control. Scale bars, 100 μm.

**Figure S9**

**
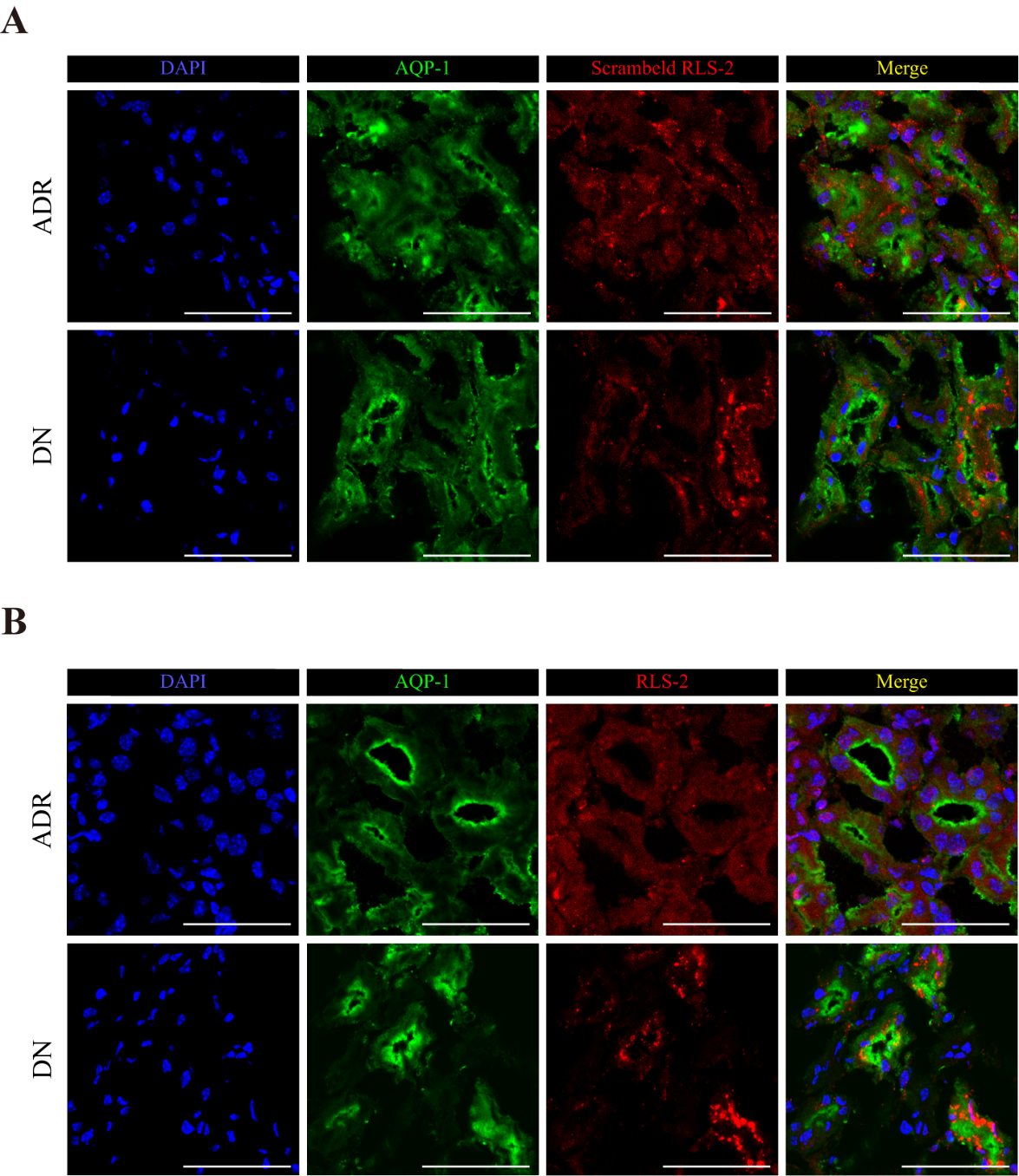
**

**Distribution of Aptamer Within Tubular Cells.** Representative confocal images show the distribution of Scrambled RLS-2 or RLS-2 (red) and the proximal tubular marker AQP1 (green). Scale bars, 50 μm.

**Figure S10**

**
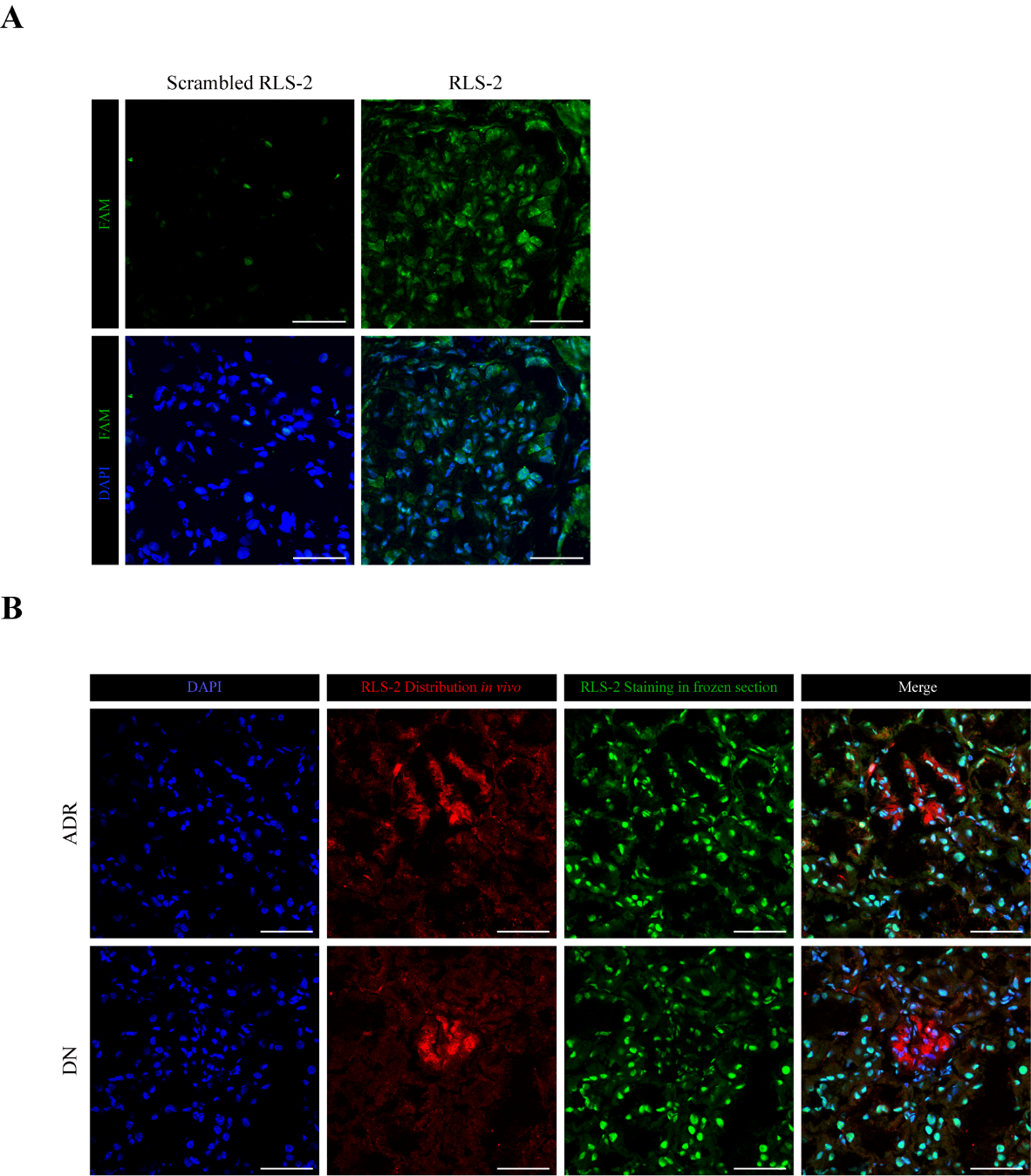
**

**Nuclear Binding of RLS-2 in Frozen Sections. (A)** Representative images of human kidney sections stained with Scrambled RLS-2 and RLS-2. Scale bars, 50 μm. **(B)** Representative confocal microscopy images showing RLS-2 distribution *in vivo* (red) and RLS-2 staining in frozen section (green). Nuclei were counterstained with DAPI (blue). Scale bars, 50 μm.

**Figure S11**


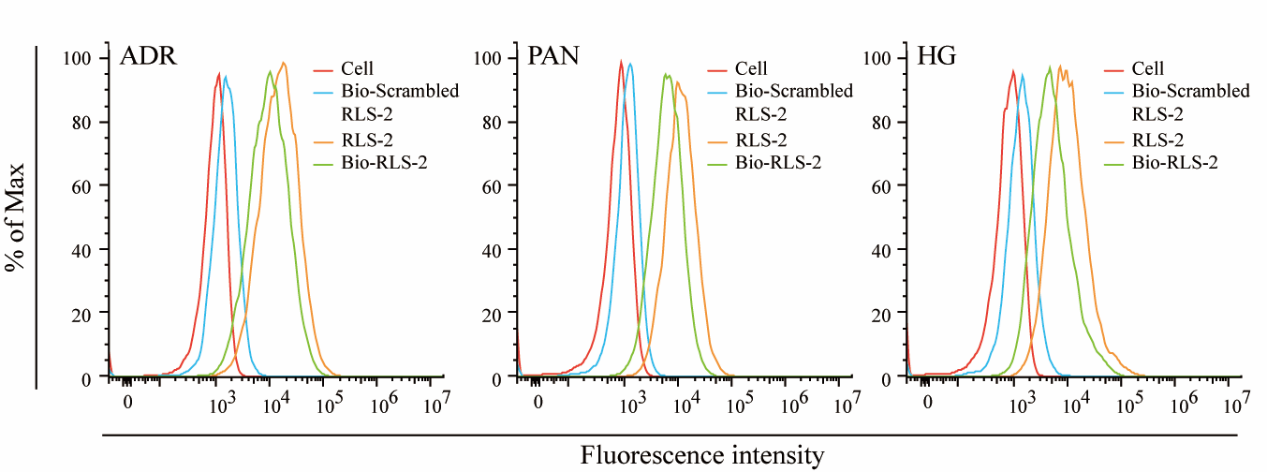


**Biotin Modification Does Not Affect the Binding of RLS-2 to Injured Podocytes.** Injured MPC5 cells were detached using 0.02% EDTA and incubated with RLS-2 or Bio-RLS-2 at 4°C for 1 hour. Bio-Scrambled RLS-2 or untreated cells were used as controls.

**Figure S12**


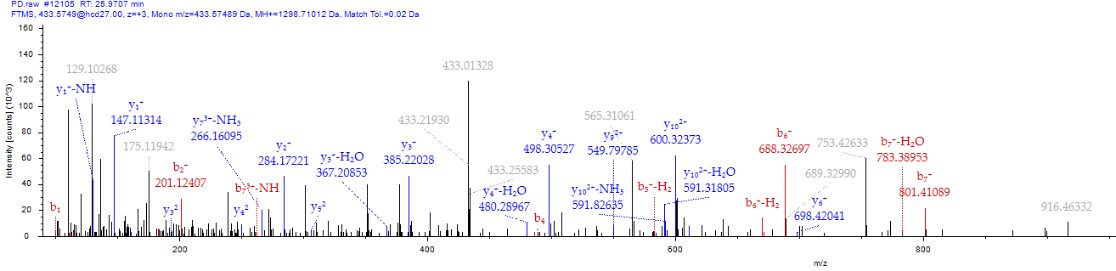


**MS/MS Spectrum of EPB41L5**

**Figure S13**


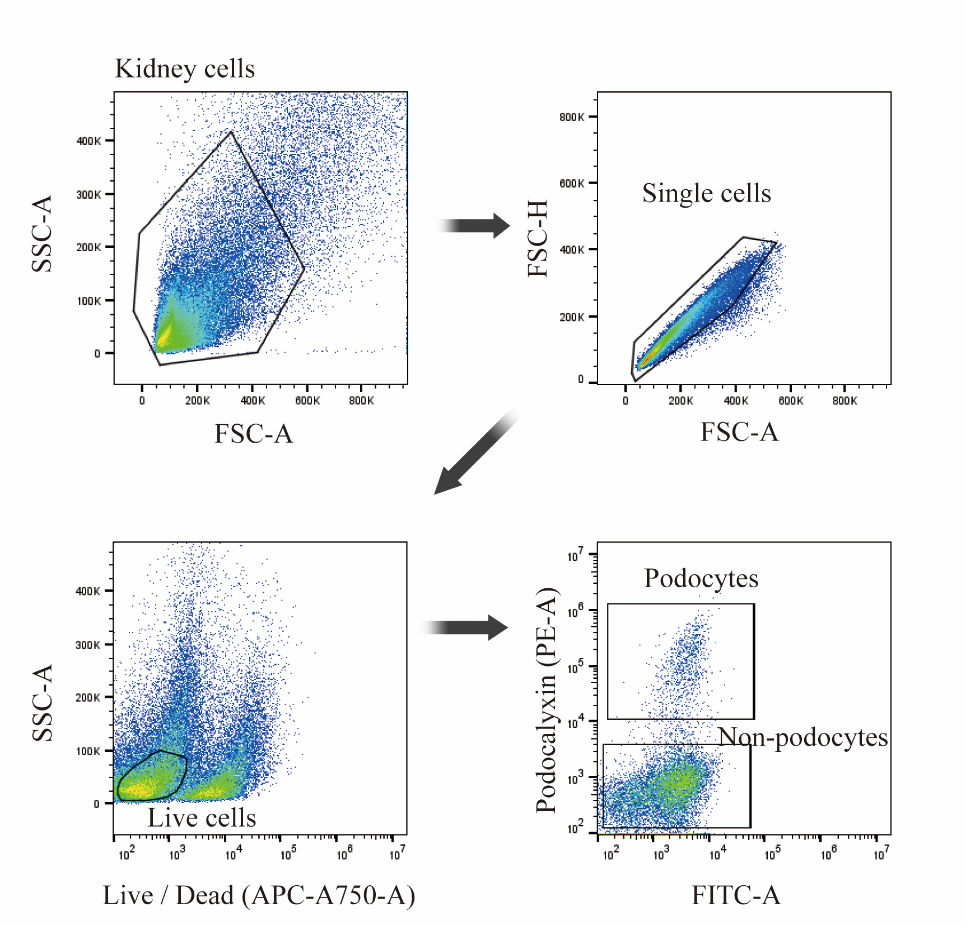


**Representative Gating Strategy for Podocytes.** Aggregated cells were excluded by gating forward scatter area (FSC-A) versus forward scatter height (FSC-H). After gating for live cells, Podocalyxin-positive cells were identified as podocytes, while the remaining cells were classified as non-podocytes.
